# Small extracellular vesicle‐derived miR‐574‐5p regulates PGE2‐biosynthesis via TLR7/8 in lung cancer

**DOI:** 10.1002/jev2.12143

**Published:** 2021-10-01

**Authors:** Julia Donzelli, Eva Proestler, Anna Riedel, Sheila Nevermann, Brigitte Hertel, Andreas Guenther, Stefan Gattenlöhner, Rajkumar Savai, Karin Larsson, Meike J. Saul

**Affiliations:** ^1^ Department of Biology Technische Universität Darmstadt Darmstadt Germany; ^2^ Department of Internal Medicine Member of the German Centre for Lung Research (DZL) Member of Cardio‐Pulmonary Institute (CPI) Justus Liebig University Giessen Germany; ^3^ Department of Pathology Justus Liebig University Giessen Germany; ^4^ Department of Lung Development and Remodelling Member of the DZL Member of CPI Max Planck Institute for Heart and Lung Research Bad Nauheim Germany; ^5^ Lung Microenvironmental Niche in Cancerogenesis Institute for Lung Health (ILH) Justus Liebig University Giessen Germany; ^6^ Rheumatology Unit Department of Medicine Karolinska University Hospital Stockholm Sweden

**Keywords:** extracellular vesicles (sEV), lung cancer, microsomal prostaglandin E synthase 1 (mPGES‐1), miR‐574‐5p, prostaglandin E2 (PGE2), toll‐like receptor 7/8 (TLR7/8)

## Abstract

Intercellular communication plays an essential role in lung cancer (LC). One of the major players in cell‐cell‐communication is small extracellular vesicles (sEV). SEV trigger various biological responses by transporting cellular cargo to target cells. One essential sEV component are microRNAs (miRs), whose transport has recently attracted increasing research interest. We report that prostaglandin E_2_ (PGE_2_), a key inflammatory lipid mediator, specifically induces the sorting of miR‐574‐5p in sEV of A549 and 2106T cells. We found that sEV‐derived miR‐574‐5p activates Toll‐like receptors (TLR) 7/8, thereby decreasing PGE_2_‐levels. In contrast, intracellular miR‐574‐5p induces PGE_2_‐biosynthesis. Consequently, the combination of intracellular and sEV‐derived miR‐574‐5p controls PGE_2_‐levels via a feedback loop. This was only observed in adeno‐ but not in squamous cell carcinoma, indicating a cell‐specific response to sEV‐derived miRs, which might be due to unique tetraspanin compositions. Hence, we describe a novel function of miR‐574‐5p unique to adenocarcinoma. Intracellular miR‐574‐5p induces PGE_2_ and thus the secretion of sEV‐derived miR‐574‐5p, which in turn decreases PGE_2_‐biosynthesis in recipient cells.

## 1 INTRODUCTION

Lung cancer (LC) is the most common cause of cancer related death worldwide (Sung et al., 2021). Non‐small cell lung cancer (NSCLC) is the most frequent LC type and accounts for approximately 80% of all cases (Molina et al., 2008). Most NSCLCs are associated with an overexpression of prostaglandin E_2_ (PGE_2_), a bioactive lipid mediator formed by two sequential reactions. First, cyclooxygenases COX‐1 and COX‐2 convert arachidonic acid to prostaglandin H_2_ (PGH_2_). Then, PGH_2_ is processed to PGE_2_ by the terminal enzyme microsomal prostaglandin E synthase 1 (mPGES‐1) (Smith et al., 2011; Yoshimatsu et al., 2001). Several studies have observed a tumour‐promoting role for PGE_2_ (Wang & Dubois, 2010). The fact that it is capable of inducing inflammation, angiogenesis, immune suppression, and proliferation, makes it an interesting therapeutic target (Nakanishi & Rosenberg, 2013; Wang & Dubois, 2010). Inhibition of PGE_2_ production not only sensitizes cancer cells to chemotherapeutic drugs (Hanaka et al., 2009). It may also help to reduce programmed cell death protein ligand 1 (PD‐L1) expression, alleviate the immune suppression, and stimulate an anti‐tumour immune response (Prima et al., 2017). Therefore, combining standard cancer therapies with PGE_2_ inhibition poses a promising anti‐tumour treatment strategy.

Recently, we identified a novel post‐transcriptional regulation mechanism for mPGES‐1‐mediated PGE_2_ biosynthesis in NSCLC (Emmerich et al., 2020; Saul et al., 2019). We demonstrated that microRNA (miR)‐574‐5p acts as a decoy to the RNA‐binding protein CUG binding protein 1 (CUGBP1) and thus antagonizes its function as a splicing silencer (Emmerich et al., 2020; Saul et al., 2019). By preventing the binding of CUGBP1 to the mPGES‐1 3´ untranslated region (UTR), miR‐574‐5p promotes alternative splicing of mPGES‐1. These results in an mPGES‐1 3´UTR isoform with a higher translation rate, increased mPGES‐1 protein levels and PGE_2_ formation and, finally, advanced tumour growth (Saul et al., 2019).

More and more evidence points towards miR‐574‐5p as a biomarker for the detection of NSCLC: increased miR‐574‐5p plasma levels in NSCLC patients are linked to tumour progression (Foss et al., 2011; Han et al., 2020; Peng et al., 2016) and cancer cells actively secrete miR‐574‐5p in small extracellular vesicles (sEV) (Han et al., 2020). But miR‐574‐5p is more than an interesting biomarker candidate. MiR‐574‐5p‐induced lung tumour growth in a xenograft mouse model was completely blocked with the mPGES‐1 inhibitor compound III (CIII) (Leclerc et al., 2013; Saul et al., 2019). Furthermore, it is strongly overexpressed in human NSCLC where high mPGES‐1 expression correlates with a low survival rate (Saul et al., 2019). This association between miR‐574‐5p and PGE_2_ may provide an indirect method to evaluate PGE_2_ levels in NSCLC and allow the detection of LC patients who will likely benefit from PGE_2_ inhibition (Saul et al., 2019).

MiRs in the blood stream are called circulating miRs. They are characterized by a remarkable stability in body fluids and changes in miR levels are associated with various diseases including cancers (Mitchell et al., 2008). The majority of circulating miRs are selectively loaded into sEV, a class of double‐membrane vesicles of 50–150 nm in diameter, which are actively secreted into the extracellular environment by all cell types (Mir & Goettsch, 2020; Raposo & Stoorvogel, 2013). Once released, sEV are delivered to target cells where they can transfer cellular components such as proteins, lipids or miRs (Penfornis et al., 2016; Thery et al., 2002). Although the factors that control the specific sorting of miRs into sEV remain unidentified, increasing evidence points towards the direction that the loading of miRs into sEV is a regulated process (Bhome et al., 2018; Groot & Lee, 2020; Lee et al., 2019; Shurtleff et al., 2016; Temoche‐Diaz et al., 2019; Villarroya‐Beltri et al., 2013). Additionally, another factor that can influence sEV function is the method of vesicle internalization. Target cells can internalize sEV via receptor‐mediated uptake, endocytosis or membrane fusion (Mathieu et al., 2019). In this context, membrane proteins like tetraspanins are already known to play a central role in cell adhesion, membrane fusion, and protein transport (Andreu & Yáñez‐Mó, 2014). Therefore, it is likely that tetraspanins are also involved in sEV uptake and functional activity (Andreu & Yáñez‐Mó, 2014). These variable internalization mechanisms plus the signalling molecules present in sEV are the reason sEV are widely accepted as important players of intercellular communication in the tumour microenvironment and worthy of investigating (Mathieu et al., 2019; Penfornis et al., 2016; Raposo & Stoorvogel, 2013).

This study set out to further elucidate the role of miR‐574‐5p in NSCLC. We found that PGE_2_ triggers the secretion of miR‐574‐5p in sEV in A549, H1650 (lung adenocarcinoma (AC)) and 2106T (lung squamous cell carcinoma (SCC)) cells without increasing intracellular miR levels or vesicle number. In a next step, the physiological function of sEV‐derived miR‐574‐5p was analysed with an artificial sEV‐miR‐574‐5p overexpression system. We found that sEV‐derived miR‐574‐5p acts as a down‐regulator of mPGES‐1 and thus decreases PGE_2_ biosynthesis in A549 cells. This novel function stands in contrast to its intracellular role of inducing PGE_2_ biosynthesis and depends on the transmission via sEV which allows miR‐574‐5p to activate endosomal Toll‐like receptors (TLR) 7/8. Both functions taken together point towards a regulatory feedback loop of miR‐574‐5p and PGE_2_ specific to A549 but not 2106T cells. Finally, we were able to demonstrate for the first time that a miR can exert two different functions which depend on intracellular expression or transmission via sEV.

## 2 MATERIALS AND METHODS

### 2.1 Cell lines and cell culture conditions

To ensure reproducibility of the results, we adopted the main recommendations of the MISEV2018 guidelines (Thery et al., 2018) in the experimental setup. An overview of the applied major recommendations is given in Table S1.

The human lung AC cell line A549 (ATCC: CCL‐185) was cultured in Dulbecco's modified Eagle medium (DMEM, Thermo Fisher Scientific, Waltham, USA) with 10% (v/v) heat‐inactivated fetal calf serum (FCS, Sigma‐Aldrich, Darmstadt, GER), 50 mg/ml gentamycin (Merck Millipore, Darmstadt, GER), and 1 mM sodium pyruvate (ThermoFisher Scientific, Waltham, USA). The human lung SCC cell line 2106T (CLS: 300165) was cultured in a 1:2 DMEM: Ham's F12K mixture (both Thermo Fisher Scientific, Waltham, USA) supplemented with 5% (v/v) heat‐inactivated FCS, 50 mg/ml gentamycin, 0.5 mM sodium pyruvate, and 15 mM 4‐(2‐hydroxylethyl)‐1‐piperazineethanesulfonic acid (HEPES, Sigma‐Aldrich, Darmstadt, GER). The human lung AC cell line H1650 (ATCC: CRL‐5883) was cultured in RPMI‐1640 Medium (Thermo Fisher Scientific, Waltham, USA) with 10% (v/v) heat‐inactivated fetal bovine serum, 50 mg/ml gentamycin, 1 mM sodium pyruvate, 10 mM HEPES (Carl Roth, Karlsruhe, GER), and 25 mM glucose (Carl Roth, Karlsruhe, GER). The human lung fibroblast cell line HFL1 (ATCC: CCL‐153) was cultivated in Ham's F‐12K medium supplemented with 10% FCS and 50 mg/ml gentamycin. According to the MISEV2018 guidelines (Thery et al., 2018), all cell culture experiments were carried out with sEV‐depleted FCS (centrifuged at 120,000×g, 4°C for 8 h in an Optima XPN‐80 ultracentrifuge, Beckman Coulter, Brea, USA) and under standard cell culture conditions (37°C, 5% CO_2_, and 98% humidity). For sEV‐derived miR‐574‐5p measurements, cells were stimulated with 5 nM PGE_2_, 5 nM Butaprost, 5 nM Sulprostone (all Sigma‐Aldrich, Darmstadt, GER), 5 nM L‐902,688 (Cayman Chemicals, Ann Arbor, USA), or vehicle dimethyl sulfoxide (DMSO, Carl Roth, Karlsruhe, Germany) for 2 h and 8 h. MPGES‐1 activity was inhibited by stimulating cells with 10 μM CIII (Cayman Chemicals, Ann Arbor, USA) or vehicle control (DMSO) for 16 h and 24 h. Cells were stimulated with 2 μg/ml purified sEV, 100 ng/ml Resiquimod (R848, Invivogen, San Diego, USA), or 200 nM ODN 2088 Control (ODN 2087) (Miltenyi Biotec, Bergisch‐Gladbach, GER) for 24 h.

### 2.2 Spheroid cultures

Spheroid monocultures of A549 cells, 2106T cells, or co‐cultures of the cancer cells with human lung fibroblasts (HFL1) were generated with the hanging drop method (Foty, 2011). 1 × 10^4^ cells were cultivated in a 50 μl drop of culture medium containing 0.4% methylcellulose (Sigma‐Aldrich, Darmstadt, GER). Co‐cultures consisted of 66% cancer cells and 33% fibroblasts. 72 h after seeding, spheroids were stimulated with 5 ng/ml IL‐1β (PeproTech, Rocky Hill, USA) for 24 h.

### 2.3 Lung tumour tissue

Lung tumour tissue samples were collected from 16 different patients with NSCLC (nine AC and seven SCC). The study protocol for tissue donation was approved by the ethics committee (Ethik Kommission am Fachbereich Humanmedizin der Justus‐Liebig‐Universität Giessen) of the University Hospital of Giessen and Marburg (Giessen, Germany) and is in accordance with national law and with Good Clinical Practice/International Conference on Harmonization guidelines. Written informed consent was obtained from each patient or the patient's next of kin (AZ 58/15) (25–27).

#### 2.3.1 sEV purification

Directly after harvesting, cell culture supernatants were centrifuged at 2000×g and room temperature (RT) for 20 min. Afterward, 1 ml supernatant was centrifuged in an Optima™ XPN‐80 ultracentrifuge (Beckman Coulter, Brea, USA) at 21,000×g and 4°C for 1 h in a 1.5 ml polypropylene tube (Beckman Coulter, Brea, USA). The supernatant was transferred to a fresh tube and centrifuged at 100,000×g and 4°C for 1 h. Finally, the supernatant was discarded and sEV pellets were resuspended in 1× phosphate buffered saline (PBS, Gibco, Carlsbad, USA). To ensure constant sEV concentrations between different experiments (Thery et al., 2018), the quantity of sEV was measured via UV‐Vis spectroscopy at an absorption wavelength of 280 nm. Purified sEV were stored at 4°C and used for experiments within 48 h.

#### 2.3.2 sEV characterization

SEV populations were characterized (Thery et al., 2018) with the ExoView R100 platform (NanoView Biosciences, Boston, USA). Cell culture supernatants of A549 and 2106T cells were incubated and stained on tetraspanin chips (CD81, CD9, CD63, IgG control, NanoView Biosciences, Boston, USA) following the manufacturer's instructions. Briefly, unpurified cell culture supernatants were incubated on the chips for 18 h at RT. Chips were washed, blocked, and incubated with antibodies against CD9 (CF488A‐labeled), CD63 (CF647‐labeled), and CD81 (CF555‐labeled) for 1 h at RT (all NanoView Biosciences, Boston, USA). Each antibody was previously diluted 1:600 in blocking solution. Finally, chips were washed, dried, and imaged with the ExoView R100 platform. The approximate particle count after ultracentrifugation was determined by interferometry (IM) vesicle sizing and single sEV cargo staining of Syntenin‐1 using the ExoView R100 platform according to the manufacturer's instructions. IM measurement detected particles within a range of 50–200 nm. SEV numbers were determined with a Zetasizer Nano S (Malvern Panalytical, Malvern, UK). To this end, 1 ml of sEV‐containing cell culture supernatant was measured in a 2.5 ml PMMA macro cuvette (Brand, Wertheim, GER). For the measurements, the refractive index of the material was set to 1.45 with an absorption of 0.001. The refractive index of the dispersant was set to 1.345 and the viscosity to 0.94 cP in order to resemble the characteristics of cell culture supernatant. Samples were measured at 21°C for 20 s.

For transmission electron microscopy (TEM), sEV from A549 and 2106T cells were purified and resuspended in 1× PBS. Then, 15 μl of purified sEV (protein concentration ∼0.025 mg/ml) were incubated on a formvar carbon coated nickel grid (Plano, Wetzlar, GER) for 10 min at RT. Samples were fixed with 2% formaldehyde (FA; Carl Roth, Karlsruhe, GER) for 10 min and then washed 3× with Milli‐Q water (MQ) and post‐stained with aqueous uranyl acetate (4%, w/v). SEV were imaged with a Zeiss EM109 electron microscope.

### 2.4 Overexpression of miR‐574‐5p in sEV

In order to overexpress miR‐574‐5p in sEV, the XMIRXpress Lenti system (System Biosciences, Palo Alto, USA) was used. A corresponding negative control was generated with the XMIRXP‐NT system (System Biosciences, Palo Alto, USA). A549 cells were seeded at a density of 5 × 10^5^ cells/well in a 6‐well plate 24 h before transfection and were transfected with 2 μg of either miR‐574‐5p overexpression (oe) or negative control plasmid and Lipofectamine 2000 (Invitrogen, Karlsruhe, GER) according to the manufacturer's instructions. 2106T cells were seeded into 12‐well plates with a density of 1 × 10^5^ cells/well 24 h before transfection and transfected with polyethylenimine (PEI, Sigma‐Aldrich, Darmstadt, Germany). In brief, 2 μg plasmid were mixed with 100 μl DMEM. 6 μl PEI (1 g/L, pH10) were mixed with 100 μl DMEM and vortexed well. Plasmid and PEI mixes were combined, vortexed, and incubated for 15 min. Afterward, the plasmid/PEI mixture was added to the cells dropwise. Both cell lines were allowed to produce miR‐574‐5p oe or negative control sEV for at least 18 h until supernatants were harvested. The overexpression was confirmed via real‐time quantitative PCR (RT‐qPCR) analysis.

### 2.5 RNase and Triton X‐100 treatment of sEV

According to the MISEV2018 guidelines (Thery et al., 2018), purified miR‐574‐5p sEV were either treated with 2.5 U/μl RNase I (New England Biolabs, Ipswich, USA) for 20 min at 37°C or with 1% TritonX 100 (Carl Roth, Karlsruhe, GER) for 10 min at RT and then with 2.5 U/μl RNase I for 20 min at 37°C. An untreated sample was used as a reference control. NEBuffer 3 (New England Biolabs, Ipswich, USA) was added to all samples. Total RNA was isolated using the miRNeasy Mini Kit (Qiagen, Hilden, GER). After RNA extraction, the amount of miR‐574‐5p was analysed via RT‐qPCR.

### 2.6 RNA extraction

Intracellular RNA was extracted using TRIzol reagent (Thermo Fisher Scientific, Waltham, USA) and digested with Turbo DNase (Thermo Fisher Scientific, Waltham, USA) according to the manufacturer's instructions. Total RNA from sEV was extracted using the phenol/guanidinium thiocyanate (GTC)‐based extraction method as previously described (Fauth et al., 2019; Hegewald et al., 2020). The samples were spiked with 0.01 ρmol synthetic ath (*Arabidopsis thaliana*)‐miR‐159a (5 ´‐UUUGGAUUGAAGGGAGCUCUA‐3′) and 1 ρmol cel (*Caenorhabditis elegans*)‐miR‐39‐3p (5′‐UCACCGGGUGUAAAUCAGCUUG‐3′, both Sigma Aldrich, Darmstadt, GER). Synthetic miRs were used as internal standards for normalization and to enhance the RNA precipitation efficiency.

### 2.7 RT‐qPCR

Intracellular and extracellular miR RT‐qPCR analysis was performed as previously described (Fauth et al., 2019; Hegewald et al., 2020). MiR analysis of PGE_2_‐stimulated A549 cells was carried out with the miScript system (Qiagen, Hilden, GER) and the following primers: miR‐574‐5p (MS00043617), ath‐miR‐159a (MS00074871), and miR reverse transcription control (miRTC) primer (MS00000001; all Qiagen, Hilden, GER). All other samples were analysed with the miRCURY system (Qiagen, Hilden, GER) and the following primers: miR‐574‐5p (YCP0044301), ath‐miR‐159a (YCP0044303), miR‐21‐5p (YP00204230), miR‐486‐5p (YP00204001) and miR‐16‐5p (YP00205702, all Qiagen, Hilden, GER). For each primer, the primer efficiency was determined according to (Pfaffl, 2001). mRNA transcripts were analysed as previously described (Saul et al., 2019) with the following primer pairs: mPGES‐1 coding sequence (mPGES‐1 CDS fwd: 5′‐GAAGAAGGCCTTTGCCAAC‐3′; mPGES‐1 CDS rev: 5′‐CCAGGAAAAGGAAGGGGTAG‐3′), mPGES‐1 (mPGES‐1 fwd: 5′‐TCCCGGGCTAAGAATGCA‐3′; mPGES‐1 rev: 5′‐ATTGGCTGGGCCAGAATTTC‐3′), mPGES‐1 3′UTR isoform (mPGES‐1 iso fwd: 5′‐GTGCCCGTGTGTGTGTATGTGTGTGTGTGT‐3′; mPGES‐1 iso rev: 5′‐CCCAGCTGGCAGACACTTCCATTTAATGACT‐3′), CUGBP1 (CUGBP1 fwd: 5′‐AAAGTCCTCCCAGGGATGCA‐3′; CUGBP1 rev: 5′‐AGCTTCCTGTCTTCCACTGCAT‐3′), COX‐2 (COX‐2 fwd: 5′‐CCGGGTACAATCGCACTTAT‐3′; COX‐2 rev: 5′‐GGCGCTCAGCCATACAG‐3′), NOXP20 (NOXP20 fwd: 5′‐GGCAAATCTCTGCTGTCGTC‐3′; NOXP20 rev: 5′‐CCTGCTTTTTCCTTGACTGC‐3′). In all experiments, GAPDH (GAPDH fwd: 5′‐TGAGAACGGGAAGCTTGTCA‐3′; GAPDH rev: 5′‐ATCGCCCCACTTGATTTTGG‐3′) was used as the endogenous control to normalize cDNA quantities between different samples.

### 2.8 Western blotting

For Western blot analysis, cells were lysed with a tissue protein extraction reagent (T‐PER, Thermo Fisher Scientific, Waltham, USA) for 30 min on ice. Protein concentrations were determined via Bradford assay (Bio‐Rad Laboratories, Hercules, USA). Western blot analysis was performed with 20–30 μg protein as previously described (Saul et al., 2019). The membranes were incubated with specific primary antibodies against mPGES‐1 (1:200, 160140, Cayman Chemicals, Ann Arbor, USA), CUGBP1 (1:500, ab129115, Abcam, Cambridge, UK), COX‐2 (1:200, ab23672, Abcam, Cambridge, UK), GAPDH (1:1000, 2118, Cell Signalling Technology, Danvers, USA) and suitable infrared fluorescent secondary antibodies (IRDye, Li‐COR Bioscience, Lincoln, USA). Western blots were visualized and analysed with the Odyssey Infrared Imaging System (LICOR Biosciences, Lincoln, USA).

### 2.9 PGE_2_ enzyme‐linked immunosorbent assay (ELISA)

For PGE_2_ measurements, cells were first stimulated with sEV for 24 h and then treated with 10 μM arachidonic acid (AA, Sigma‐Aldrich, Darmstadt, GER). 15 min after the addition of AA, supernatants were harvested and centrifuged at 17,000×g and 4°C for 5 min to eliminate cell debris. Supernatants were transferred to fresh tubes and PGE_2_ concentrations were measured using a PGE_2_ ELISA (514010, Cayman Chemicals, Ann Arbor, USA) according to the manufacturer's instructions. All samples were measured in duplicates. The absorbance was determined with the Tecan Infinite M 2000 (Tecan Group, Männedorf, CH).

### 2.10 Tetrazolium reduction assay

Cell proliferation and metabolism were assessed with a 3‐(4,5‐dimethylthiazol‐2‐yl)‐2,5‐diphenyltetrazoliumbromide (MTT) (Carl Roth, Karlsruhe, GER) reduction assay. Cells were seeded at a density of 1 × 10^4^ cells/well in a 96‐well plate and treated with 2 μg/ml sEV (as described above) 24 h after seeding. After 24 h, cells were incubated with MTT (5 mg/ml in cell culture medium) for 3 h at 37°C. The medium was removed and 100 μl DMSO/well were added. The quantity of formazan was measured with the Tecan Infinite M 2000 plate reader at an absorption wavelength of 570 nm. A reference measurement at 630 nm was performed to subtract nonspecific background.

### 2.11 Immunohistochemistry (IHC) staining of paraffin‐embedded tissue sections

Paraffin‐embedded NSCLC tissue sections were deparaffinized 3× for 5 min in xylene (Carl Roth, Karlsruhe, GER) and rehydrated in a sequential ethanol (EtOH, VWR, Radnor, USA) series (100%, 96% and 70% (v/v) EtOH) for 5 min in each bath. Afterward, samples were incubated in MQ 3× for 5 min. Antigens were unmasked by boiling the samples in 1× citrate buffer (10 mM trisodium citrate, pH6, Carl Roth, Karlsruhe, GER) 3× for 5 min. Samples were allowed to cool for 30 min, washed with 1× PBS 3× for 3 min and blocked with 3% BSA (Sigma‐Aldrich, Darmstadt, GER) in 1× PBS (w/v) for 30 min. Tissues were incubated with α‐mPGES‐1 or α‐CUGBP1 antibody diluted in blocking solution (1:200, 160140, Cayman Chemicals, Ann Arbor, USA; 1:100, ab129115, Abcam, Cambridge, UK) at 4°C overnight. Then, samples were washed with 0.01% Tween20 (Carl Roth, Karlsruhe, GER) in 1× PBS (v/v) (PBST) 3× for 5 min. Endogenous peroxidases were blocked by incubation with 3% H_2_O_2_ (Sigma‐Aldrich, Darmstadt, GER) in 1x PBS (v/v) for 10 min. Tissues were washed with 1× PBS 3× for 3 min and incubated with an HRP‐labelled secondary antibody diluted in blocking solution (1:300, α‐rabbit‐HRP, ab7090, Abcam, Cambridge, UK) for 45 min. After washing with PBST 3× for 5 min, the staining was developed with a 3,3′‐Diaminobenzidine (DAB) substrate kit (Abcam, Cambridge, UK) according to the manufacturer's instructions. Samples were counterstained with haematoxylin (Sigma‐Aldrich, Darmstadt, GER) for 20 s and washed under running tap water for 10 min. Finally, samples were dehydrated in a sequential EtOH series (70%, 96% and 100% (v/v) EtOH) for 1 min each, briefly incubated in xylene and mounted in EUKITT (VWR, Radnor, USA). DAB development time was controlled on an unstained tissue section. Stained tissue sections were imaged with the Aperio CS2 slide scanner (Leica, Wetzlar, GER).

### 2.12 In situ hybridization (ISH) of paraffin‐embedded tissue sections

Tissue sections were deparaffinized and rehydrated as described above and then incubated in 1× PBS for 5 min. Tissues were permeabilized with 3 μg/ml proteinase K (Qiagen, Hilden, GER) at 37°C for 10 min, washed in 1× PBS twice for 3 min and prehybridized in ISH buffer (Qiagen, Hilden, GER) for 20 min at 50°C. Afterwards, samples were hybridized with 100 nM of a double‐fluorescein (FAM) labelled locked nucleic acid (LNA) miRNA probe (Qiagen, Hilden, GER) against miR‐574‐5p at 54°C for 1 h. Stringent washes were carried out in saline‐sodium citrate buffer (SSC, Invitrogen, Karlsruhe, GER) at hybridization temperature as follows: 5 min in 5× SSC, 2 × 5 min in 1× SSC and 2 × 5 min in 0.2× SSC. Then, the samples were washed in 0.2× SSC for 5 min and in 1× PBS for 5 min at RT. Tissues were blocked in 1x PBS containing 0.1% Tween20, 2% sheep serum (Jackson Immunoresearch, Ely, UK), and 1% BSA for 15 min. Afterwards, samples were incubated with an alkaline phosphatase (AP)‐labelled antibody against FAM (11 426 338 910, Roche, Basel, CHE) diluted 1:500 in 1× PBS with 0.05% Tween20, 1% sheep serum and 1% BSA for 1 h. Samples were washed in PBST 3× for 3 min and in 1× PBS 1× for 3 min. Staining was developed at 30°C for 2 h with nitro blue tetrazolium/5‐bromo‐4‐chloro‐3‐indolyl‐phosphate (NBT‐BCIP, Roche, Basel, CHE) as a substrate in MQ containing 0.2 mM levamisole (Sigma‐Aldrich, Darmstadt, GER) to block endogenous phosphatases. The substrate reaction was stopped by incubation in KTBT buffer (50 mM Tris‐HCL, 150 mM NaCl, 10 mM KCl, all Carl Roth, Karlsruhe, GER) 2× for 5 min and an additional incubation in tap water for 5 min. Finally, sections were counterstained with nuclear fast red (Vector Laboratories, Burlingame, USA) for 60 s, washed under running tap water for 10 min and dehydrated and mounted as described above. A digoxygenin (DIG)‐labelled scramble control probe (Qiagen, Hilden, GER) was used in combination with an AP‐labelled antibody against DIG (1:500, 11 093 274 910, Roche, Basel, CHE) to control for unspecific binding. Stained tissue sections were imaged with the Aperio CS2 slide scanner.

### 2.13 Immunofluorescence

2.5 × 10^5^ cells/well were seeded on glass coverslips (12 mm; Neolab, Heidelberg, Germany) in a 6‐well plate. After 24 h, cells were fixed with 4% FA for 10 min. Samples were washed with 1× PBS 3× for 3 min, permeabilized with 0.5% Triton X‐100 in 1× PBS for 10 min and washed again with 1× PBS 3× for 3 min. Cells were blocked with 4% BSA (w/v) (Sigma Aldrich, Darmstadt, GER) in 1× PBS for 20 min. The primary antibodies against TLR7 (MA5‐16247, Invitrogen, Thermo Fisher Scientific, Waltham, USA) and TLR8 (MA5‐16190, Invitrogen, Thermo Fisher Scientific, Waltham, USA) were diluted 1:50 (TLR7) or 1:500 (TLR8) in blocking solution. Cells were incubated with primary antibodies for 1 h at RT. Afterwards, cells were washed with PBST 3× for 5 min and incubated with a secondary antibody (ab150116, Abcam, Cambridge, UK), diluted 1:500 in blocking solution, for 45 min at RT. Cells were washed with PBST 3× for 5 min, counterstained with 4′,6‐diamidino‐2‐phenylindole (DAPI; Sigma Aldrich, Darmstadt, GER) for 5 min, and washed 3× for 3 min in 1x PBS. Finally, cells were mounted in Mowiol 4–88 (Sigma Aldrich, Darmstadt, GER) mounting medium containing 2% DABCO (Sigma Aldrich, Darmstadt, GER). Images were acquired with a Leica TCS SPE confocal point scanner which was mounted on a Leica DMi8 stand and equipped with an oil immersion 60X Apochromat (Leica Microsystems, Buffalo Grove, USA). Images were processed with the ImageJ software (National Institutes of Health, Bethesda, USA; http://imagej.nih.gov/ij/) and show one focal plane within the middle of the nucleus.

### 2.14 Live cell imaging

Cells were seeded into 8‐well chamber slides (IBIDI, Gräfelfing, GER) at a density of 2.6 × 10^4^ cells/well. After 24 h, nuclei were stained with 5 μg/ml Hoechst 33258 (Sigma‐Aldrich, Darmstadt, GER) for 30 min at 37°C. Previously purified sEV were stained with the lipophilic tracer 3,3′‐dioctadecyloxacarbocy‐anine perchlorate (DiO, Sigma‐Aldrich, Darmstadt, GER) for 15 min at 37°C. Cells were imaged in 5 min intervals over 35 min and stained sEV were added after the first image was taken. Imaging was carried out using an UltraVIEW VoX spinning disk system (PerkinElmer, Waltham, USA) mounted on a Nikon TI microscope (Nikon, Minato, Japan) or with a Nikon Eclipse Ti and equipped with a climate chamber (37°C, 5% CO_2_, 60% humidity). Images were acquired with a cooled 14‐bit EMCCD camera (1000 × 1000‐pixel frame transfer EMCCD, 30 fps at full frame 1× 1 binning 35 MHz readout, 8 × 8 μm pixel size) using Volocity 6.3 (PerkinElmer, Waltham, USA) or a Nikon DS‐Qi2 camera and NIS elements software (Nikon, Minato, Japan). Cells were imaged by taking z intervals of 2 μm with a 60× objective, which were then merged into one image using the ImageJ software (http://imagej.nih.gov/ij/).

### 2.15 Statistics

Results are shown as the mean + standard error of the mean (SEM) of at least three independent experiments. Statistical analysis was carried out by Student's unpaired t‐test (two‐tailed) or one‐way ANOVA with Tukey's multiple comparison test using GraphPad Prism 6.0. Experimental differences were considered as significant for *P*≤0.05 (indicated as * for *P*≤0.05, ** for *P*≤0.005, *** for *P*≤0.001, and **** for *P*≤0.0001, or § for *P*≤0.05, §§§ for *P*≤0.001, and §§§§ for *P*≤0.0001).

## 3 RESULTS

### 3.1 MiR‐574‐5p and mPGES‐1 colocalize in human NSCLC tissue

**FIGURE 1:**
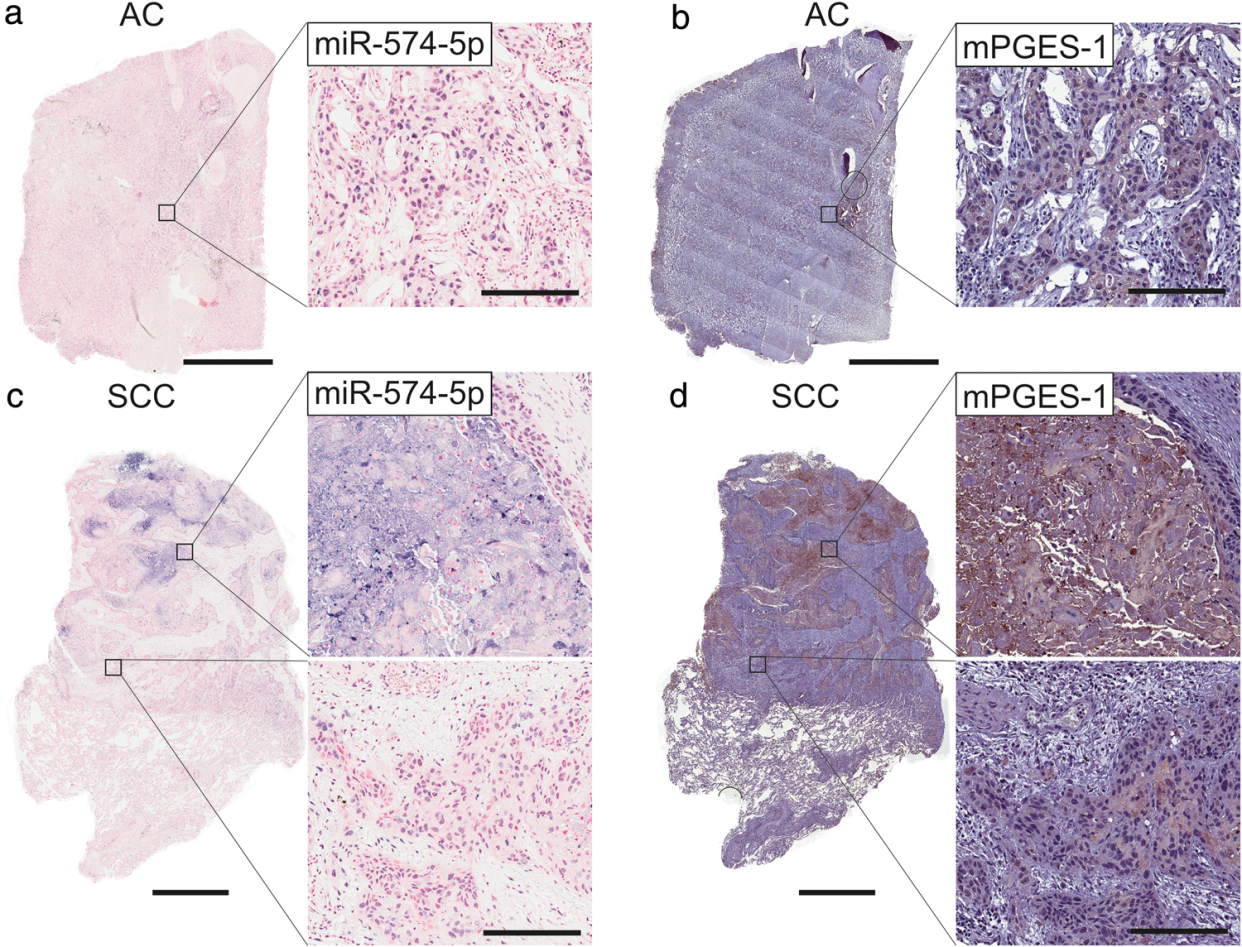
MiR‐574‐5p and mPGES‐1 colocalize in human lung cancer. Paraffin‐embedded tissue sections were stained for miR‐574‐5p (blue) via in situ hybridization (ISH) and for mPGES‐1 (brown) via immunohistochemistry (IHC). (a) AC tissue section stained for miR‐574‐5p, counterstained with nuclear fast red (red). (b) AC tissue section stained for mPGES‐1, counterstained with haematoxylin (blue). (c) SCC tissue section stained for miR‐574‐5p, counterstained with nuclear fast red (red). (d) SCC tissue section stained for mPGES‐1, counterstained with haematoxylin (blue). Representative images of 14 independent patients (7x AC and 7X SCC) are shown. Scale bars in AC tissue sections: 3 mm, SCC tissue sections: 2 mm, magnified images: 200 μm

To compare PGE_2_‐biosynthesis in AC and SCC and to analyse if the miR‐574‐5p/CUGBP1 decoy mechanism is present in NSCLC tissue, we stained human lung tissue sections of AC and SCC patients for miR‐574‐5p and mPGES‐1. To this end, miR‐574‐5p was visualized via ISH using complementary LNA‐probes and mPGES‐1 via IHC with a specific antibody. The use of serial sections allowed a comparison of all staining within the same tumour sample. In AC tissue sections, miR‐574‐p and mPGES‐1 were localized within NSCLC cells. MiR‐574‐5p was detected in the nuclei, whereas mPGES‐1 was present in the cytoplasm of cancer cells (Figure 1a, b). In SCC sections, miR‐574‐5p and mPGES‐1 expression were much higher compared to AC. Both were present within the cancer cells. Like AC samples, miR‐574‐5p was located inside the nuclei and mPGES‐1 in the cytoplasm. In addition, miR‐574‐5p and mPGES‐1 were strongly expressed in inflamed/necrotic areas of the tissue (Figure 1c, d), which was not observed in AC samples (Figure S1A, B). Staining controls did not show specific signals for miR‐574‐5p or mPGES‐1 (Figure S1C). It is important to note that negative control ISH was performed on a tissue section from the same SCC tumour sample used for miR‐574‐5p ISH. Additionally, CUGBP1 was stained via IHC and detected in the nuclei of cancer cells in AC and SCC, although it was not present in inflamed/necrotic areas of SCC (Figure S1D, E). CUGBP1 and mPGES‐1 primary antibodies were of the same isotype. The comparison of both staining confirms that both antibodies specifically bound their target. Our results show that the interaction partners of the miR‐574‐5p/CUGBP1 decoy mechanism are present within the same cells in NSCLC tissue. Thus, they can theoretically interact with each other in NSCLC cells in order to modulate PGE_2_‐biosynthesis.

In order to further analyse PGE_2_‐biosynthesis in both tumour types, we generated spheroid mono‐ and co‐cultures of A549 (AC) and 2106T (SCC) cells with human lung fibroblasts (Figure S2). We chose the 3D model system over conventional 2D cell culture since it more closely mimics in vivo tumour characteristics such as nutrient gradients and cellular interactions. Also, it was shown that spheroid cultures constitute a more physiological model to study PGE_2_ regulation (Kock et al., 2020). In general, 2106T spheroids revealed higher protein and mRNA levels of mPGES‐1 and COX‐2 than A549 spheroids (Figure S2A, B, S3A‐D). While IL‐1β stimulation was capable of inducing mPGES‐1 only in A549 spheroids, COX‐2 was induced in spheroids of both cell types. There were no differences in CUGBP1 or miR‐574‐5p levels between both cell types (Figure S2C, D). But miR‐574‐5p levels were slightly decreased with IL‐1β stimulation and further decreased upon co‐cultivation with fibroblasts in all spheroids (Figure S2D). In 2106T spheroids, decreased miR‐574‐5p levels did not coincide with a lower expression of nervous system overexpressed protein 20 (NOXP20), which carries the precursor of miR‐574‐5p in its intron (Zhang et al., 2014) (Figure S3F). Since NOXP20 mRNA levels usually correlate with miR‐574‐5p levels, this suggests that other factors, like an increase in miR‐574‐5p secretion, may be responsible for the reduced levels in 2106T spheroids.

In summary, our results confirm that A549 and 2106T cells both express the interaction partners of the miR‐574‐5p/CUGBP1 decoy mechanism. However, differences in the expression profile and inducibility of PGE_2_‐synthesizing proteins indicate a cell‐type‐specific regulation of PGE_2_‐biosynthesis.

### 3.2 A549‐ and 2106T‐derived sEV differ in their tetraspanin composition

**FIGURE 2:**
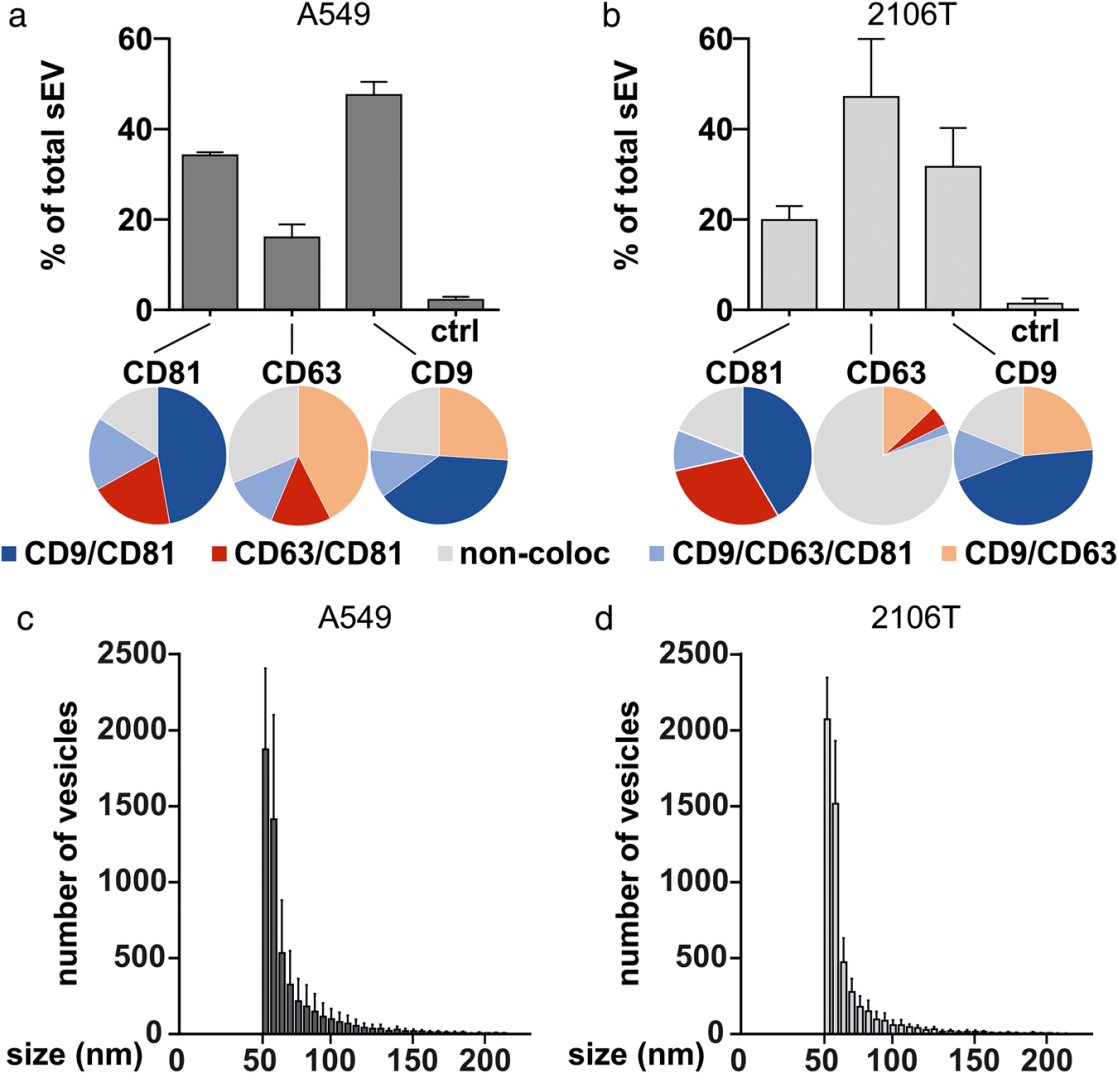
A549‐ and 2106T‐derived sEV differ in their tetraspanin composition. (a, b) Unpurified A549‐ and 2106T‐derived sEV were analysed using the ExoView R100 platform (NanoView Biosciences). SEV were captured at specific antibody‐coated spots against CD81, CD63, and CD9 (bar graphs). Captured sEV were further analysed for tetraspanin colocalization with specific fluorescent antibodies (pie charts). (c, d) The size distribution of A549‐ and 2106T‐derived sEV was analysed using the ExoView R100 platform. Data are presented as mean +SEM of two independent biological experiments with three technical replicates

In order to further characterize the observed differences between A549 and 2106T cells, we next focused on the role of sEV in cell‐cell‐communication, which is a crucial factor in the progression of NSCLC (Zheng et al., 2018). To this end, we analysed sEV of A549 and 2106T cells using the ExoView R100 platform. This system allows the characterization of unpurified sEV, which is a great advantage because manipulation of sEV populations during the purification process is avoided (Bachurski et al., 2019; Daaboul et al., 2016). In our analysis, we focused on the characterization of the tetraspanin composition. The ExoView R100 platform can identify colocalized tetraspanins on a single sEV level by measuring CD81, CD63, and CD9 in the same sample. We observed that A549‐ and 2106T‐derived sEV strongly differed in their CD63 levels (Figure 2a, b). While most 2106T‐derived sEV were bound to the CD63 spots (47% of total sEV), only 16% of total A549‐derived sEV were bound to CD63 spots. In 2106T‐derived sEV, the CD81 spots showed the lowest number with 20% of total sEV. In contrast, most A549‐derived sEV were bound to the CD9 spot with 47% of total sEV. Next, we analysed the colocalization of CD81, CD63 and CD9 (Figure 2a, b, pie charts). CD81 and CD9 positive sEV showed very similar colocalization patterns in both cell types. In contrast, CD63 positive sEV revealed cell‐specific differences. In 43% of A549‐derived CD63 positive sEV, CD9 and CD63 colocalized. 14% showed colocalization of CD81 and CD63, while 12% of sEV were positive for all three tetraspanins. 31% of CD63 positive A549‐derived sEV did not colocalize with other tetraspanins. In contrast, in 2106T‐derived sEV, 80% were only positive for CD63. 13% of CD63 positive 2106T‐derived sEV showed colocalization of CD9 and CD63, while 5% were positive for CD81 and CD63. Only 2% of all CD63 positive 2106T‐derived sEV were positive for all three tetraspanins. These results show that A549‐ and 2106T‐derived sEV greatly differ in their tetraspanin composition, which might influence the interaction between these sEV and possible target cells.

Finally, we analysed the size distribution of A549‐ and 2106T‐derived sEV (Figure 2c, d). Both sEV types had a small number of sEV with a diameter of 100–200 nm. Most sEV had a diameter of 50 nm, which also marks the detection limit of the ExoView R100 platform. Therefore, it is likely that A549 and 2106T cells also secrete sEV smaller than 50 nm, which were not detected with this approach. Taken together, our results indicate that A549‐ and 2106T‐derived sEV did not differ in size distribution but showed a different tetraspanin composition.

The unique tetraspanin composition of A549‐ and 2106T‐derived sEV constitutes another crucial difference between both cell types, which could have a great influence on the interaction with target cells.

### 3.3 PGE_2_ induces the secretion of miR‐574‐5p in sEV

**FIGURE 3:**
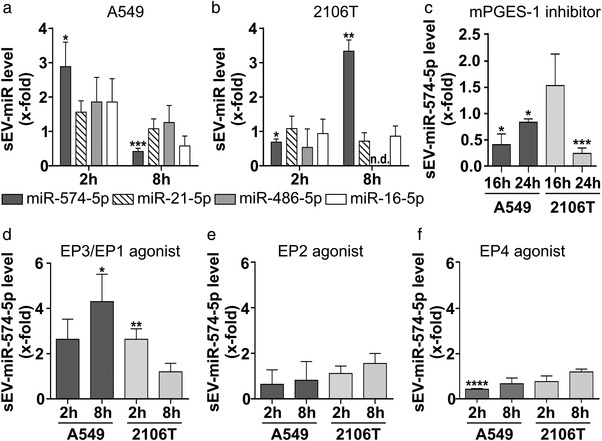
PGE_2_ induces the secretion of sEV‐derived miR‐574‐5p in human lung cancer cells. (a, b) SEV‐miR level of miR‐574‐5p, ‐21‐5p, 486‐5p, and 16‐5p isolated from A549 (*N* = 4‐5) and 2106T (N = 3) cell culture supernatants. A549 and 2106T cells were stimulated with 5 nM PGE_2_ for 2 h and 8 h prior to harvesting. (c) SEV‐derived miR‐574‐5p levels of A549 and 2106T cells after treatment with 10 μM CIII (mPGES‐1 inhibitor, *N* = 3‐4). (d‐f) SEV‐derived miR‐574‐5p of A549 and 2106T cells stimulated with 5 nM EP receptor agonist Butaprost (N = 4), Sulprostone (*N* = 3), and L‐902,688 (*N* = 3). SEV‐derived RNA was analysed via RT‐qPCR, normalized to the spike‐in control ath‐miR‐159a and folded to their corresponding vehicle control. Data are shown as mean +SEM. Unpaired t‐test, *P≤0.05; **P≤0.01, ***P≤0.001; ****P≤0.0001

Tissue staining and spheroid cultures demonstrated that miR‐574‐5p and mPGES‐1 are localized in NSCLC cells and that PGE_2_‐biosynthesis is specifically regulated in A549 and 2106T cells. Moreover, we showed that A549‐ and 2106T‐derived sEV had unique tetraspanin compositions. Thus, we aimed to further analyse the sEV secretion in both cell types. Since it is known that PGE_2_ is overexpressed in many NSCLC cases, we stimulated A549 and 2106T cells with 5 nM PGE_2_ to mimic the inflammatory environment of the tumour. After 2 and 8 h, we purified sEV from cell culture supernatants using differential ultracentrifugation and isolated the total RNA of sEV. MiR‐574‐5p, ‐21‐5p, ‐486‐5p, and ‐16‐5p levels were quantified via RT‐qPCR. These miRs are known to exert physiological functions in LC and are discussed as functional biomarkers (Foss et al., 2011; Han et al., 2020; Liu et al., 2017; Reis et al., 2020; Tian et al., 2019). We found that only miR‐574‐5p was significantly enriched in A549‐ and 2106T‐derived sEV after PGE_2_ stimulation (Figure 3a, b). A549‐derived sEV showed a significant 3‐fold increase in miR‐574‐5p 2 h after stimulation (Figure 3a). 8 h after PGE_2_ treatment, A549 cells secreted sEV with reduced miR‐574‐5p content. Like A549 cells, 2106T cells also secreted miR‐574‐5p in sEV in response to PGE_2_. However, we observed the highest level of miR‐574‐5p after 8 h, not after 2 h as in A549 cells (Figure 3b). In addition, we measured miR‐574‐5p secretion in another NSCLC cell line in response to PGE_2_ stimulation. The results showed that the H1650 cell line was also prone to miR‐574‐5p secretion 8 h after stimulation (Figure S4A).

Finally, the secretion of other miRs was not significantly affected by PGE_2_ stimulation in 2106T and A549 cells. This is particularly interesting since the basal level of miR‐574‐5p is generally lower than that of the other miRs in both cell lines (Figure S4B, C).

We next examined whether the increase in sEV‐derived miR‐574‐5p was associated with a higher expression of miR‐574‐5p (Figure S4D, E). We did not observe a significant increase in intracellular miR‐574‐5p after PGE_2_ stimulation in A549 or 2106T cells. Thus, the increased secretion of miR‐574‐5p was not caused by higher intracellular expression levels of miR‐574‐5p.

To test whether the increase in sEV‐derived miR‐574‐5p correlated with higher sEV numbers or higher miR‐574‐5p levels per vesicle, we measured sEV numbers using a Zetasizer Nano S (Figure S4F, G). There were no significant changes in sEV numbers in response to PGE_2_ treatment which suggests that miR content per sEV was increased.

Additionally, we blocked endogenous PGE_2_‐biosynthesis with the mPGES‐1 inhibitor CIII (Foss et al., 2011). Both cell lines showed a significant decrease in sEV‐derived miR‐574‐5p upon mPGES‐1 inhibition (Figure 3c). These results indicate that both external stimulation with PGE_2_ and intracellular PGE_2_‐synthesis regulate miR‐574‐5p secretion. To assess which PGE_2_ receptor (EP receptor) induces miR‐574‐5p secretion after PGE_2_ stimulation, we treated A549 and 2106T cells with EP receptor agonists. Stimulation with EP1/EP3 agonists resulted in increased secretion of miR‐574‐p in A549 and 2106T cells (Figure 3d), while EP2 and EP4 agonist stimulation did not (Figure 3e, f). Here, we observed different secretion time points compared to PGE_2_ stimulation, which is probably linked to different binding affinities and component stabilities of the agonists and PGE_2_.

In sum, our results strongly indicate that PGE_2_ rapidly and specifically induces the sorting of miR‐574‐5p into sEV of A549 and 2106T cells via EP1/3 receptors.

### 3.4 Overexpression of miR‐574‐5p in A549‐ and 2106T‐derived sEV

**FIGURE 4:**
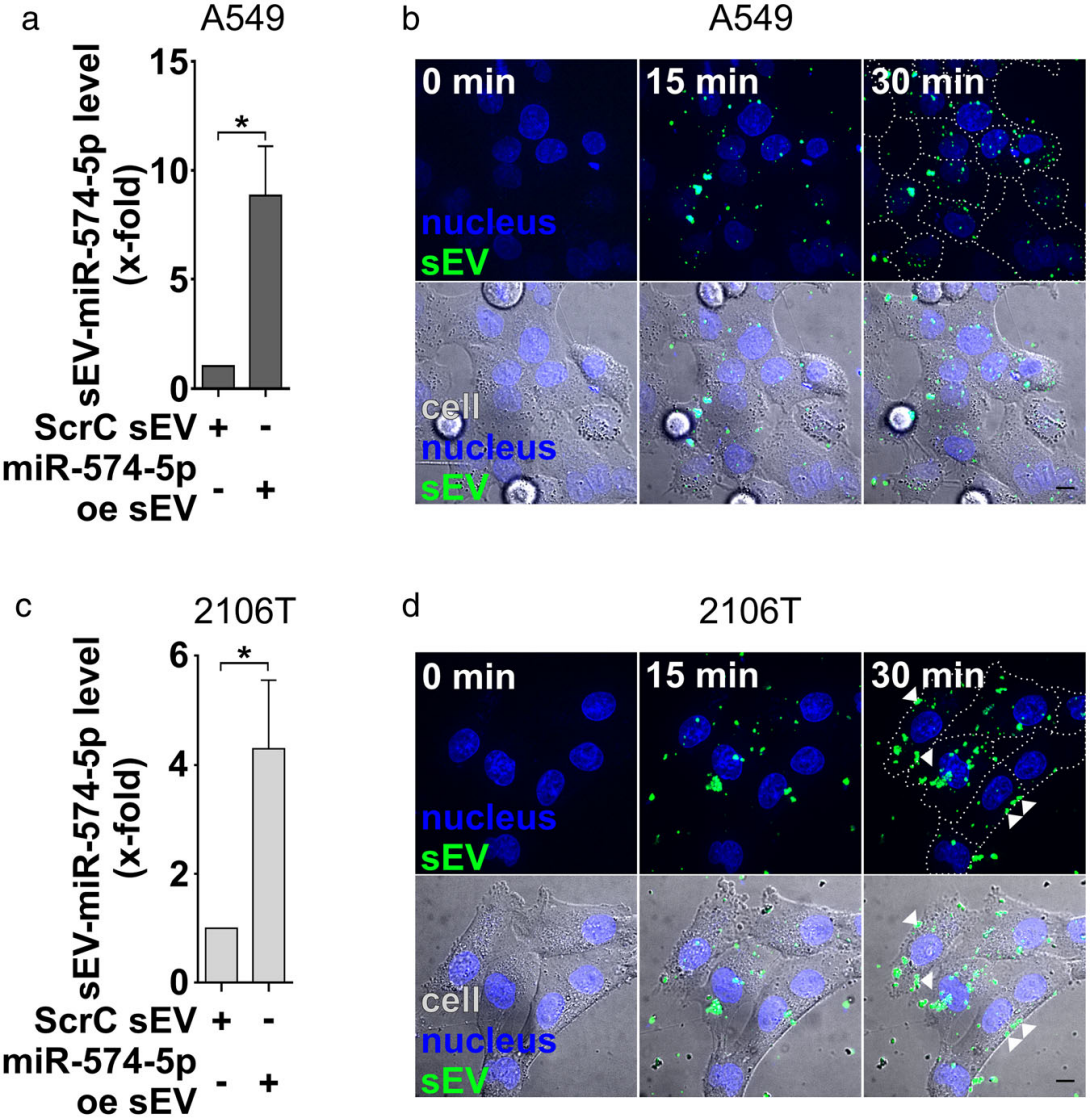
Characterization of A549‐ and 2106T‐derived miR‐574‐5p oe sEV. (A, C) A549 and 2106T cells were transfected with scrambled control (ScrC) or miR‐574‐5p overexpression (miR‐574‐5p oe) XMIRXpress constructs (System Bioscience). MiR‐574‐5p levels were analysed via RT‐qPCR, normalized to the spike‐in control ath‐miR‐159a and folded to their corresponding negative control (A549: *N* = 3, 2106T: *N* = 4). Relative changes to ScrC control are shown as mean + SEM, unpaired t‐test *p≤0.05. (B, D) Live cell imaging of miR‐574‐5p oe sEV uptake in A549 and 2106T cells. Cells were treated with their respective miR‐574‐5p oe sEV. Cells were visualized using differential interference contrast (DIC, grey) and nuclear staining with 5 μg/ml Hoechst 33258 (blue). SEV were stained with the lipophilic tracer 3,3′‐dioctadecyloxacarbocy‐anine perchlorate DIO (green). SEV uptake was imaged for 30 min. Some 2106T sEV accumulated at the cell membrane (D, white arrows). Representative images of three independent biologicals replicates with at least three technical replicates are shown. Scale bars: 10 μm

To analyse the physiological function of sEV‐derived miR‐574‐5p, we established an overexpression system for both cancer cell lines as previously established in HEK‐293 cells (Hegewald et al., 2020). The system increases the loading of miR‐574‐5p into sEV (miR‐574‐5p oe sEV). Control experiments were performed with sEV loaded with a scrambled miR (ScrC sEV). We compared miR‐574‐5p levels of both sEV types via RT‐qPCR and detected a ∼9‐fold increase of miR‐574‐5p in A549‐derived oe sEV (Figure 4a). 2106T‐derived oe sEV showed a ∼4‐fold increase of miR‐574‐5p compared to ScrC sEV (Figure 4b). We purified sEV via differential ultracentrifugation and confirmed the successful isolation of sEV with TEM analysis (Figure S5A). To determine the number of sEV isolated after ultracentrifugation, we performed a representative analysis of oe sEV of 2106T cells with ExoView R100 and determined an approximate particle count of 30,000 vesicles per ml of supernatant (Figure S5B).

To verify whether miR‐574‐5p was loaded into sEV and not attached to the outside, we performed an exemplary RNase protection experiment with A549‐ and 2106T‐derived miR‐574‐5p oe sEV as we had done previously (Shurtleff et al., 2016). For this purpose, sEV were treated with RNase alone or in combination with a detergent, followed by RT‐qPCR analysis (Figure S5C, D). We showed that the majority of miR‐574‐5p was protected within sEV with engineered miR‐574‐5p level. The disruption of sEV followed by an RNase digest led to a significant decrease in miR‐574‐5p levels. Hence, miR‐574‐5p was protected in samples without detergent, indicating that most of the miRs were located within sEV.

Finally, we showed that miR‐574‐5p oe sEV were taken up by A549 and 2106T cells using live cell imaging (Figure 4b, d). Both cell types started to internalize sEV within a few minutes. While most A549‐derived sEV were distributed within the whole cell, some 2106T‐derived sEV seemed to accumulate at the cell membrane (Figure 4d, white arrows). To find out whether the accumulation of sEV at cell membranes was vesicle‐ or cell‐specific, 2106T and A549 cells were also treated with sEV of the other cell line. When 2106T cells were incubated with A549‐derived sEV, an increased accumulation of sEV at the cell membrane was observed 30 min after addition of the sEV (Figure S6A). In contrast, A549 cells appeared to take up 2106T‐derived sEV (S6B). This suggests a cell‐specific interaction between cells and sEV.

Either way, we were able to show that both cell lines internalize engineered sEV, which enables a physiological interaction. To control for cytotoxic effects of miR‐574‐5p oe sEV, we performed a tetrazolium reduction assay (Figure S7A, B). To this end, A549 and 2106T cells were stimulated with miR‐574‐5p oe and ScrC sEV. 24 h after sEV treatment, we did not observe any impact on cell viability or cell metabolism caused by treatment with miR‐574‐5p oe sEV. We thus conclude that we successfully established cell‐specific miR‐574‐5p oe and control sEV to analyse the physiological role of sEV‐derived miR‐574‐5p. These engineered sEV are internalized by both cell types and do not exert cytotoxic effects on cells.

### 3.5 MiR‐574‐5p oe sEV decrease mPGES‐1 and PGE_2_‐levels via TLR7/8 in A549 cells

After establishing an overexpression system for miR‐574‐5p in NSCLC‐derived sEV, we analysed the physiological function of sEV‐derived miR‐574‐5p. Because miR‐574‐5p can activate TLR7/8 signalling (Fabbri et al., 2012; Hegewald et al., 2020), we included a TLR7/8 ligand R848 and inhibitor ODN 2088 Control (ODN 2087) in our experiments. A549 and 2106T cells were treated with either self‐derived miR‐574‐5p oe or ScrC sEV, or a TRL7/8 ligand for 24 h. All conditions were also controlled by blocking TLR7/8 activation with a TLR7/8 inhibitor.

**FIGURE 5:**
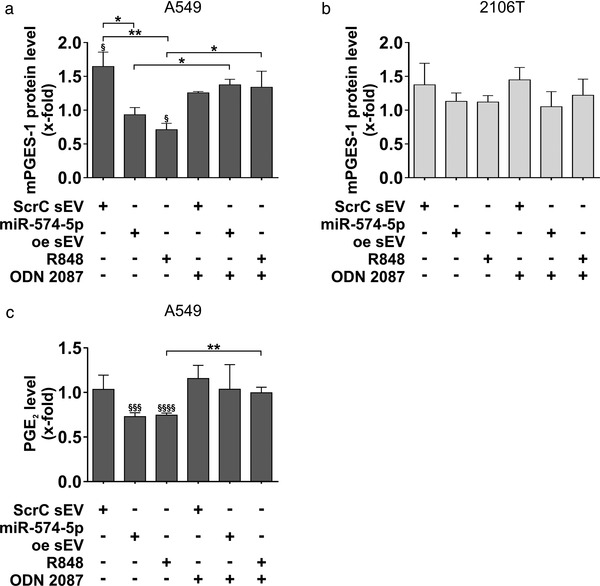
SEV‐derived miR‐574‐5p decreases mPGES‐1 and PGE_2_ via TLR7/8 activation in A549 cells. (a, b) Western blot analysis of A549 and 2106T cells treated with 2 μg/ml sEV, 100 ng/ml R848 (TLR7/8 ligand) or 200 mM ODN 2088 Control (ODN 2087) (TLR7/8 antagonist). Cells were treated for 24 h. MPGES‐1 levels were normalized to GAPDH and folded to untreated cell samples (A549: *N* = 3‐5, 2106T: *N* = 5‐6). (c) PGE_2_‐levels of A549 cells. 24 h after stimulation, cells were treated with 10 μM arachidonic acid (AA) for 15 min. PGE_2_‐levels were measured via an enzyme‐linked immunosorbent assay (Cayman Chemicals, *N* = 4). Results are shown as mean +SEM, unpaired t‐test to other samples, *P≤0.05; **P≤0.01; ***P≤0.001; ****P≤0.0001. Unpaired t‐test to untreated control § *P*≤0.05; §§§ *P*≤0.001; §§§§ *P*≤0.0001

After 24 h, we analysed mPGES‐1 protein levels by Western blot analysis. In A549 cells, ScrC sEV led to a significant 1.6‐fold increase in mPGES‐1 protein expression compared to the untreated control (Figure 5a and Figure S7D). In contrast, stimulation with miR‐574‐5p oe sEV led to a slight decrease by 0.9‐fold. Compared to ScrC sEV treated samples, miR‐574‐5p oe sEV significantly decreased mPGES‐1 protein levels by 0.5‐fold. Treatment with the TLR7/8 ligand resulted in significantly lower mPGES‐1 levels of 0.7‐fold compared to ScrC sEV‐treated samples and the untreated control. Blocking TLR7/8 receptors significantly prevented the mPGES‐1 decrease induced by miR‐574‐5p oe sEV or R848. Overall, these results indicate that activation of TLR7/8 via sEV‐derived miR‐574‐5p regulates mPGES‐1 in A549 cells. Moreover, regulation of mPGES‐1 also affected PGE_2_‐levels (Figure 5c). We observed significantly lower PGE_2_‐levels in A549 cells treated with miR‐574‐5p oe sEV or R848. Administration of the TLR7/8 inhibitor also blocked this downregulation.

Because miR‐574‐5p is a major post‐transcriptional regulator of PGE_2_‐biosynthesis, we measured intracellular miR‐574‐5p levels after treatment with miR‐574‐5p oe sEV (Figure S7C). As expected, we observed an increase in intracellular miR‐574‐5p in response to sEV uptake 1 h after treatment. Interestingly, intracellular miR‐574‐5p was significantly decreased 3 h after sEV treatment compared to the 1 h values and the untreated control. These results suggest that intracellular miR‐574‐5p levels are also regulated by sEV‐derived miR‐574‐5p. As observed for mPGES‐1 regulation, this may also be due to the activation of TLR7/8 in A549 cells.

In contrast to A549 cells, we did not observe significant effects in 2106T cells using the same experimental setup (Figure 5b, Fig. S7E). 2106T cells did not show regulation of mPGES‐1 upon stimulation with either the TLR7/8 ligand R848 or the TLR7/8 antagonist ODN 2088 Control (ODN 2087). Thus, our results indicate that A549 and 2106T cells respond cell‐specifically to sEV‐derived miR‐574‐5p. While miR‐574‐5p oe sEV activated TLR7/8 signalling in A549 cells and thus regulated mPGES‐1‐dependent PGE_2_‐biosynthesis, this effect was not observed in 2106T cells. Similar results were shown with the TLR7/8 ligand. Taken together, these results indicate that 2106T cells do not respond to TLR7/8 activation as A549 cells do.

## 4 DISCUSSION

A better understanding of intercellular communication in the tumour microenvironment is a valuable basis for developing new cancer treatment approaches. Recent studies identified sEV as key players in processes that contribute to disease development (Mathieu et al., 2019). This is mainly mediated by sEV cargo. One major cargo is miRs, which are highly associated with the hallmarks of cancer (Bhome et al., 2018; Garzon et al., 2006). In addition, it is well known that tumour development can also be induced by inflammatory mediators (Mantovani et al., 2008). But the interaction between inflammatory signalling and sEV‐derived miRs in the tumour microenvironment has not yet been studied. A key inflammatory mediator in cancer development is PGE_2_, which promotes tumour progression, immune evasion, and angiogenesis (Wang & Dubois, 2010). In this study, we demonstrate that PGE_2_ significantly induces sEV‐miR‐574‐5p secretion in A549, 2106T and H1650 cells. This is of particular interest because intracellular miR‐574‐5p regulates mPGES‐1‐dependent PGE_2_‐biosynthesis in lung AC (Emmerich et al., 2020; Saul et al., 2019). This increased secretion of sEV‐derived miR‐574‐5p was not caused by higher intracellular miR‐574‐5p levels, nor by increased sEV numbers. These results demonstrate for the first time that PGE_2_ specifically triggers the sorting of a miR, here miR‐574‐5p, into sEV and that this sorting is mediated by EP1 and EP3 receptors.

Several studies show that increased miR‐574‐5p plasma levels of LC patients correlate with tumour development (Foss et al., 2011; Han et al., 2020; Peng et al., 2016). Particularly interesting is the fact that the level of circulating sEV‐derived miR‐574‐5p is not only elevated in LC patients compared to healthy controls, but also significantly decreases after tumour resection (Han et al., 2020). Taken together with the fact that miR‐574‐5p specifically regulates PGE_2_‐biosynthesis in NSCLC (Emmerich et al., 2020; Saul et al., 2019), miR‐574‐5p can be considered as a promising candidate for a minimally invasive biomarker for the stratification of NSCLC patients (Saul et al., 2019). We showed that the localization of miR‐574‐5p and mPGES‐1 in NSCLC tumour tissues correlated and that AC and SCC differ in the expression of miR‐574‐5p and mPGES‐1 in tumour tissues and 3D tumour models. Furthermore, PGE_2_‐induced secretion of miR‐574‐5p was significantly reduced by a PGE_2_ inhibitor. These results support the hypothesis that the level of extracellular miR‐574‐5p correlates with the level of PGE_2_ in the tumour. This will not only enable an initial patient classification, but also the monitoring of treatment success. Hence, the suitability of miR‐574‐5p as biomarker for NSCLC should be further investigated in clinical studies.

Next, we analysed cell‐specific sEV characteristics. Our results revealed a unique tetraspanin composition on the surface of sEV for A549 and 2106T cells. Other studies already showed that the tetraspanin composition on sEV probably influences the selection of target cells and the mechanism of sEV uptake (Horibe et al., 2018; Rana et al., 2012). Also, the expression of surface proteins and receptors on the cell membrane strongly influences sEV uptake. Since the composition of membrane proteins is unique to different cell types, cell‐specific sEV uptake is likely (Jadli et al., 2020). Taken together, the membrane characteristics of sEV and recipient cells allow cell type‐specific responses to sEV. We speculate that A549 and 2106T cells respond differently to sEV because some 2106T‐derived sEV accumulated at the cell membrane. Thus, specific interactions with sEV and different uptake mechanisms can be expected.

In addition to this, we aimed to understand the physiological function of sEV‐derived miR‐574‐5p. Several studies already describe the physiological function of sEV‐derived miRs, although the physiological relevance is still under discussion (Bayraktar et al., 2017; Chevillet et al., 2014). Functional analysis of sEV‐derived miR‐574‐5p was performed with miR‐574‐5p oe sEV (Hegewald et al., 2020). Surprisingly, in A549 cells, miR‐574‐5p oe sEV led to a significant decrease of mPGES‐1 and PGE_2_. Recently, miR‐574‐5p was described as a ligand for TLR7/8. It was shown that miR‐574‐5p directly interacts with immune receptors with a high binding affinity (Fabbri et al., 2012; Hegewald et al., 2020). In rheumatoid arthritis, osteoclast maturation is promoted by sEV‐derived miR‐574‐5p via the activation of TLR7/8 signalling without altering PGE_2_‐biosynthesis (Hegewald et al., 2020). Here, we demonstrated that a TLR7/8 antagonist inhibited the miR‐574‐5p‐mediated decrease of PGE_2_‐biosynthesis in A549 cells. In contrast, like miR‐574‐5p oe sEV, a TLR7/8 ligand decreased PGE_2_‐synthesis.

Hence, we describe a novel function for miR‐574‐5p, which is dependent on the transfer via sEV. So far, it was not clear whether sEV only transfer miRs or also impact their physiological function. We describe that intracellular and sEV‐derived miR‐574‐5p exerts opposing functions on the PGE_2_‐biosynthesis. Depending on the uptake mechanism, an sEV‐derived miR can be released at different locations within the cell (McKelvey et al., 2015). In A549 cells, sEV‐derived miR‐574‐5p can interact with the endosomal TLR7/8 receptors and thus decrease PGE_2_‐levels. Consequently, sEV transfer miR‐574‐5p to a novel subcellular location which is not reached by intracellular miR‐574‐5p. This demonstrates that sEV not only act as miR shuttles, but are also capable of changing their mode of action. Contrary to A549 cells, we did not observe the described mechanism in 2106T cells. In these cells, sEV‐derived miR‐574‐5p did not affect PGE_2_‐synthesis, although 2106T cells express TLR7/8 similarly to A549 cells (Figure S7F, G). The lack of this regulatory mechanism could explain the higher mPGES‐1 levels in 2106T spheroid cultures compared to A549 spheroids. The fact that sEV‐derived miR‐574‐5p does not exert an autocrine function on 2106T PGE_2_‐biosynthesis, supports our initial theory of cell‐specific interactions with sEV. This is consistent with our live cell experiments which indicate a cell‐type‐specific sEV uptake and with other studies describing cell‐type‐specific uptake mechanisms for similar sEV (Horibe et al., 2018). Furthermore, it is conceivable that 2106T‐derived sEV physiologically target other recipient cells rather than interacting with their donor cell. Specific targeting of recipient cells has been shown previously for other cell types (Denzer et al., 2000; Mallegol et al., 2007). Here, we show that A549‐ and 2106T‐derived sEV interact differently with their donor cells and elicit different physiological responses when it comes to mPGES‐1‐dependent PGE_2_‐biosynthesis. The observation that sEV‐derived miR‐574‐5p decreases PGE_2_‐levels in A549 cells stands in contrast to other studies with immune cells that showed increased PGE_2_‐synthesis after TLR7/8 activation (Hattermann et al., 2007; Salvi et al., 2016). We attribute this discrepancy mainly to cell‐specific effects. One possible downstream signalling pathway after TLR7/8 activation in A549 cells is nuclear factor kappa‐light‐chain‐enhancer of activated B cells (NFκB) (Dowling, 2018). Interestingly, NFκB‐p65, a subunit of the NFκB transcription complex, significantly reduces intracellular miR‐574‐5p (Ku et al., 2017). NFκB‐p65 can regulate transcription within 30 min after activation (Zambrano et al., 2014). This is in line with our results. We demonstrated that sEV‐derived miR‐574‐5p reduces intracellular miR‐574‐5p expression. Therefore, we hypothesize that sEV‐derived miR‐574‐5p acts as a TLR7/8 ligand and activates NFκB signalling which results in decreased intracellular miR‐574‐5p and PGE_2_‐levels. This assumption supports the hypothesis that TLR7/8 activation has antitumoral effects (Michaelis et al., 2019; Schön & Schön, 2008).

In sum, we demonstrated that PGE_2_ modulates the sorting of miR‐574‐5p into sEV of A549 and 2106T cells. Furthermore, we analysed the physiological function of sEV‐derived miR‐574‐5p in recipient cells and showed that it decreases mPGES‐1‐dependent PGE_2_‐biosynthesis in A549, but not in 2106T cells. This cell‐specific PGE_2_ regulation is of great interest for the development of new therapeutic approaches in the treatment of NSCLC. The benefits of PGE_2_ inhibitors for the treatment of LC are well known. Our results can contribute to the development of new subtype‐specific therapies based on a more efficient use of PGE_2_ inhibitors. However, the results of this study should be characterized in additional AC and SCC cell lines, primary cancer cells, and in vivo models. Future studies need to further investigate the relationship between sEV uptake and the physiological function of sEV in the tumour microenvironment to unravel the therapeutic potential of our findings.

**FIGURE 6:**
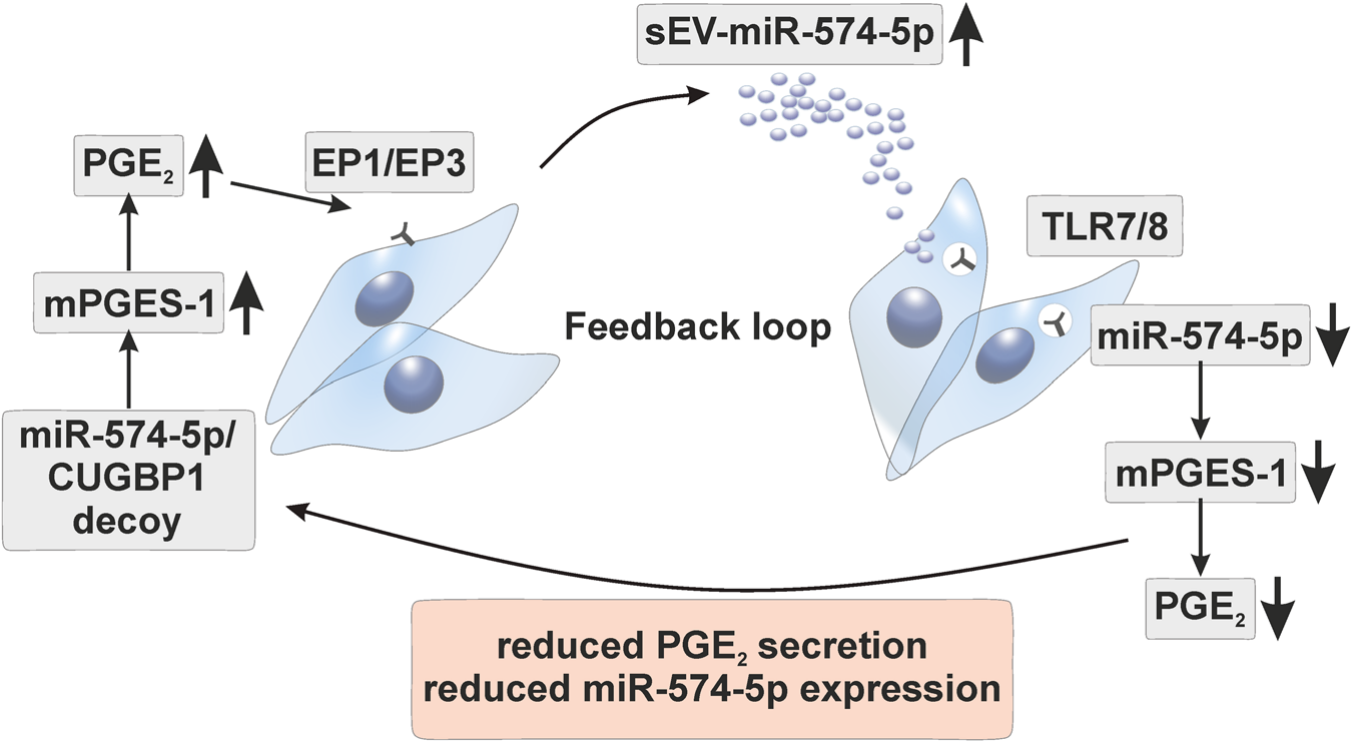
Schematic diagram of the PGE_2_ feedback loop regulation mediated by miR‐574‐5p and TLR7/8. The miR‐574‐5p/CUGBP1 decoy regulates PGE_2_‐biosynthesis intracellularly. High intracellular miR‐574‐5p induces PGE_2_‐biosynthesis. PGE_2_ triggers the secretion of miR‐574‐5p in sEV. In recipient A549 cells, sEV‐derived miR‐574‐5p activates TLR7/8 signalling, which leads to decreased miR‐574‐5p, mPGES‐1 and PGE_2_‐levels

Finally, while intracellular miR‐574‐5p induces PGE_2_‐biosynthesis via a decoy mechanism with CUGBP1, sEV‐derived miR‐574‐5p exerts opposite functions on the regulation of PGE_2_. This novel mechanism is mediated by the transfer of miR‐574‐5p via sEV, which enables an interaction with endosomal TLR7/8. Hence, the interplay between intracellular and sEV‐derived miR‐574‐5p controls PGE_2_‐levels in A549 cells via a feedback loop (Figure 6). This study demonstrates for the first time that the same miR can have different functions depending on whether it is expressed inside a cell or transferred to a cell via sEV.

## CONFLICT OF INTEREST

The authors declare that the research was conducted in the absence of any commercial or financial relationships that could be construed as a potential conflict of interest.

## FUNDING

This project was supported by BMBF‐KMU‐innovativ‐22: miRTumorProst (161B0768B), Wilhelm Sander‐Foundation (2018.115.1), Athene Young Investigator program (Technische Universität Darmstadt; grant no.: n/a), LOEWE‐Zentrum Translationale Medizin und Pharmakologie (TP‐18).

## AUTHOR CONTRIBUTIONS

Julia Donzelli and Eva Proestler performed the experiments and analysed the data. Julia Donzelli wrote the manuscript. Eva Proestler contributed to manuscript writing. Anna Riedel performed spheroid experiments and supported secretion experiments. Sheila Nevermann performed RNase protection assay experiments, supported secretion experiments and contributed to manuscript writing. Brigitte Hertel performed the TEM analysis. Rajkumar Savai, Andreas Guenther and Stefan Gattenlöhner provided human lung tissue samples. Rajkumar Savai and Karin Larsson supported the tissue staining experiments. Meike J. Saul conceived the study, designed, and supervised the overall project, and wrote the manuscript. All authors conducted the quality assurance of the paper and reviewed the manuscript.

## Supporting information

Supporting Information

## References

[jev212143-bib-0001] Andreu, Z., & Yáñez‐Mó, M. (2014). Tetraspanins in extracellular vesicle formation and function. Frontiers in Immunology, 5, 442. 25278937 10.3389/fimmu.2014.00442 PMC4165315

[jev212143-bib-0002] Bachurski, D., Schuldner, M., Nguyen, P. H., Malz, A., Reiners, K. S., Grenzi, P. C., Babatz, F., Schauss, A. C., Hansen, H. P., Hallek, M., & Pogge von Strandmann, E. (2019). Extracellular vesicle measurements with nanoparticle tracking analysis ‐ an accuracy and repeatability comparison between NanoSight NS300 and ZetaView. Journal Extracellular Vesicles, 8, 1596016. 10.1080/20013078.2019.1596016 PMC6450530 30988894

[jev212143-bib-0003] Bayraktar, R., Van Roosbroeck, K., & Calin, G. A. (2017). Cell‐to‐cell communication: microRNAs as hormones. Molecular Oncology, 11, 1673–1686. 29024380 10.1002/1878-0261.12144 PMC5709614

[jev212143-bib-0004] Bhome, R., Del Vecchio, F., Lee, G. H., Bullock, M. D., Primrose, J. N., Sayan, A. E., & Mirnezami, A. H. (2018). Exosomal microRNAs (exomiRs): small molecules with a big role in cancer. Cancer Letters, 420, 228–235. 29425686 10.1016/j.canlet.2018.02.002 PMC5831981

[jev212143-bib-0005] Chevillet, J. R., Kang, Q., Ruf, I. K., Briggs, H. A., Vojtech, L. N., Hughes, S. M., Cheng, H. H., Arroyo, J. D., Meredith, E. K., Gallichotte, E. N., Pogosova‐Agadjanyan, E. L., Morrissey, C., Stirewalt, D. L., Hladik, F., Yu, E. Y., Higano, C. S., & Tewari, M. (2014). Quantitative and stoichiometric analysis of the microRNA content of exosomes. PNAS, 111, 14888–14893. 25267620 10.1073/pnas.1408301111 PMC4205618

[jev212143-bib-0006] Daaboul, G. G., Gagni, P., Benussi, L., Bettotti, P., Ciani, M., Cretich, M., Freedman, D. S., Ghidoni, R., Ozkumur, A. Y., Piotto, C., Prosperi, D., Santini, B., Ünlü, M. S., & Chiari, M. (2016). Digital detection of exosomes by interferometric imaging. Scientific Reports, 6, 37246. 27853258 10.1038/srep37246 PMC5112555

[jev212143-bib-0007] Denzer, K., van Eijk, M., Kleijmeer, M. J., Jakobson, E., de Groot, C., & Geuze, H. J. (2000). Follicular dendritic cells carry MHC class II‐expressing microvesicles at their surface. Journal of Immunology, 165, 1259–1265. 10.4049/jimmunol.165.3.1259 10903724

[jev212143-bib-0008] Dowling, D. J. (2018). Recent advances in the discovery and delivery of TLR7/8 agonists as vaccine adjuvants. Immunohorizons, 2, 185–197. 31022686 10.4049/immunohorizons.1700063

[jev212143-bib-0009] Emmerich, A. C., Wellstein, J., Ossipova, E., Baumann, I., Lengqvist, J., Kultima, K., Jakobsson, P. J., Steinhilber, D., & Saul, M. J. (2020). Proteomics‐based characterization of miR‐574‐5p decoy to CUGBP1 suggests specificity for mPGES‐1 regulation in human lung cancer cells. Front Pharmacology, 11, 196. 10.3389/fphar.2020.00196 PMC7082395 32231562

[jev212143-bib-0010] Fabbri, M., Paone, A., Calore, F., Galli, R., Gaudio, E., Santhanam, R., Lovat, F., Fadda, P., Mao, C., Nuovo, G. J., Zanesi, N., Crawford, M., Ozer, G. H., Wernicke, D., Alder, H., Caligiuri, M. A., Nana‐Sinkam, P., Perrotti, D., & Croce, C. M. (2012). MicroRNAs bind to toll‐like receptors to induce prometastatic inflammatory response. PNAS, 109, E2110–6. 22753494 10.1073/pnas.1209414109 PMC3412003

[jev212143-bib-0011] Fauth, M., Hegewald, A. B., Schmitz, L., Krone, D. J., & Saul, M. J. (2019). Validation of extracellular miRNA quantification in blood samples using RT‐qPCR. FASEB BioAdvances, 1, 481–492. 32123845 10.1096/fba.2019-00018 PMC6996320

[jev212143-bib-0012] Foss, K. M., Sima, C., Ugolini, D., Neri, M., Allen, K. E., & Weiss, G. J. (2011). miR‐1254 and miR‐574‐5p: Serum‐based microRNA biomarkers for early‐stage non‐small cell lung cancer. Journal of Thoracic Oncology: Official Publication of the International Association for the Study of Lung Cancer, 6, 482–488. 10.1097/JTO.0b013e318208c785 21258252

[jev212143-bib-0013] Foty, R. (2011). A simple hanging drop cell culture protocol for generation of 3D spheroids. Journal of Visualized Experiments: JoVE, (51), e2720. 10.3791/2720 PMC3197119 21587162

[jev212143-bib-0014] Garzon, R., Fabbri, M., Cimmino, A., Calin, G. A., & Croce, C. M. (2006). MicroRNA expression and function in cancer. Trends in Molecular Medicine, 12, 580–587. 17071139 10.1016/j.molmed.2006.10.006

[jev212143-bib-0015] Groot, M., & Lee, H. (2020). Sorting mechanisms for MicroRNAs into extracellular vesicles and their associated diseases. Cells, 9. 10.3390/cells9041044 PMC7226101 32331346

[jev212143-bib-0016] Han, Z., Li, Y., Zhang, J., Guo, C., Li, Q., Zhang, X., Lan, Y., Gu, W., Xing, Z., Liang, L., Li, M., & Mi, S. (2020). Tumor‐derived circulating exosomal miR‐342‐5p and miR‐574‐5p as promising diagnostic biomarkers for early‐stage Lung Adenocarcinoma. International Journal of Medical Sciences, 17, 1428–1438. 32624699 10.7150/ijms.43500 PMC7330662

[jev212143-bib-0017] Hanaka, H., Pawelzik, S. C., Johnsen, J. I., Rakonjac, M., Terawaki, K., Rasmuson, A., Sveinbjornsson, B., Schumacher, M. C., Hamberg, M., Samuelsson, B., Jakobsson, P. J., Kogner, P., & Radmark, O. (2009). Microsomal prostaglandin E synthase 1 determines tumor growth in vivo of prostate and lung cancer cells. PNAS 106, 18757–18762. 19846775 10.1073/pnas.0910218106 PMC2768589

[jev212143-bib-0018] Hattermann, K., Picard, S., Borgeat, M., Leclerc, P., Pouliot, M., & Borgeat, P. (2007). The Toll‐like receptor 7/8‐ligand resiquimod (R‐848) primes human neutrophils for leukotriene B4, prostaglandin E2 and platelet‐activating factor biosynthesis. Faseb Journal, 21, 1575–1585. 17264163 10.1096/fj.06-7457com

[jev212143-bib-0019] Hegewald, A. B., Breitwieser, K., Ottinger, S. M., Mobarrez, F., Kortokova, M., Rethi, B., Jakobsson, P. J., Catrina, A. I., Wähämaa, H., & Saul, M. J. (2020). Extracellular miR‐574‐5p induces osteoclast differentiation via TLR 7/8 in rheumatoid arthritis. Front Immunology, 11, 585282. 10.3389/fimmu.2020.585282 PMC7591713 33154755

[jev212143-bib-0020] Horibe, S., Tanahashi, T., Kawauchi, S., Murakami, Y., & Rikitake, Y. (2018). Mechanism of recipient cell‐dependent differences in exosome uptake. Bmc Cancer [Electronic Resource], 18, 47. 10.1186/s12885-017-3958-1 PMC5756423 29306323

[jev212143-bib-0021] Jadli, A. S., Ballasy, N., Edalat, P., & Patel, V. B. (2020). Inside(sight) of tiny communicator: exosome biogenesis, secretion, and uptake. Molecular and Cellular Biochemistry, 467, 77–94. 32088833 10.1007/s11010-020-03703-z

[jev212143-bib-0022] Kock, A., Bergqvist, F., Steinmetz, J., Elfman, L. H. M., Korotkova, M., Johnsen, J. I., Jakobsson, P. J., Kogner, P., & Larsson, K. (2020). Establishment of an in vitro 3D model for neuroblastoma enables preclinical investigation of combined tumor‐stroma drug targeting. Faseb Journal, 34, 11101–11114. 32623799 10.1096/fj.202000684R

[jev212143-bib-0023] Ku, T., Li, B., Gao, R., Zhang, Y., Yan, W., Ji, X., Li, G., & Sang, N. (2017). NF‐κB‐regulated microRNA‐574‐5p underlies synaptic and cognitive impairment in response to atmospheric PM(2.5) aspiration. Particle and Fibre Toxicology, 14, 34. 10.1186/s12989-017-0215-3 28851397 PMC5575838

[jev212143-bib-0024] Leclerc, P., Idborg, H., Spahiu, L., Larsson, C., Nekhotiaeva, N., Wannberg, J., Stenberg, P., Korotkova, M., & Jakobsson, P. J. (2013). Characterization of a human and murine mPGES‐1 inhibitor and comparison to mPGES‐1 genetic deletion in mouse models of inflammation. Prostaglandins & Other Lipid Mediators, 107, 26–34. 24045148 10.1016/j.prostaglandins.2013.09.001

[jev212143-bib-0025] Lee, H., Li, C., Zhang, Y., Zhang, D., Otterbein, L. E., & Jin, Y. (2019). Caveolin‐1 selectively regulates microRNA sorting into microvesicles after noxious stimuli. Journal of Experimental Medicine, 216, 2202–2220. 10.1084/jem.20182313 PMC6719430 31235510

[jev212143-bib-0026] Liu, Q., Yu, Z., Yuan, S., Xie, W., Li, C., Hu, Z., Xiang, Y., Wu, N., Wu, L., Bai, L., & Li, Y. (2017). Circulating exosomal microRNAs as prognostic biomarkers for non‐small‐cell lung cancer. Oncotarget, 8, 13048–13058. 28055956 10.18632/oncotarget.14369 PMC5355076

[jev212143-bib-0027] Mallegol, J., Van Niel, G., Lebreton, C., Lepelletier, Y., Candalh, C., Dugave, C., Heath, J. K., Raposo, G., Cerf‐Bensussan, N., & Heyman, M. (2007). T84‐intestinal epithelial exosomes bear MHC class II/peptide complexes potentiating antigen presentation by dendritic cells. Gastroenterology, 132, 1866–1876. 17484880 10.1053/j.gastro.2007.02.043

[jev212143-bib-0028] Mantovani, A., Allavena, P., Sica, A., & Balkwill, F. (2008). Cancer‐related inflammation. Nature, 454, 436–444. 18650914 10.1038/nature07205

[jev212143-bib-0029] Mathieu, M., Martin‐Jaular, L., Lavieu, G., & Théry, C. (2019). Specificities of secretion and uptake of exosomes and other extracellular vesicles for cell‐to‐cell communication. Nature Cell Biology, 21, 9–17. 30602770 10.1038/s41556-018-0250-9

[jev212143-bib-0030] McKelvey, K. J., Powell, K. L., Ashton, A. W., Morris, J. M., & McCracken, S. A. (2015). Exosomes: mechanisms of uptake. Journal Circulating Biomark, 4, 7. 10.5772/61186 PMC5572985 28936243

[jev212143-bib-0031] Michaelis, K. A., Norgard, M. A., Zhu, X., Levasseur, P. R., Sivagnanam, S., Liudahl, S. M., Burfeind, K. G., Olson, B., Pelz, K. R., Angeles Ramos, D. M., Maurer, H. C., Olive, K. P., Coussens, L. M., Morgan, T. K., & Marks, D. L. (2019). The TLR7/8 agonist R848 remodels tumor and host responses to promote survival in pancreatic cancer. Nature Communications, 10, 4682. 10.1038/s41467-019-12657-w PMC6794326 31615993

[jev212143-bib-0032] Mir, B., & Goettsch, C. (2020). Extracellular vesicles as delivery vehicles of specific cellular cargo. Cells, 9. 10.3390/cells9071601 PMC7407641 32630649

[jev212143-bib-0033] Mitchell, P. S., Parkin, R. K., Kroh, E. M., Fritz, B. R., Wyman, S. K., Pogosova‐Agadjanyan, E. L., Peterson, A., Noteboom, J., O'Briant, K. C., Allen, A., Lin, D. W., Urban, N., Drescher, C. W., Knudsen, B. S., Stirewalt, D. L., Gentleman, R., Vessella, R. L., Nelson, P. S., Martin, D. B., & Tewari, M. (2008). Circulating microRNAs as stable blood‐based markers for cancer detection. PNAS, 105, 10513–10518. 18663219 10.1073/pnas.0804549105 PMC2492472

[jev212143-bib-0034] Molina, J. R., Yang, P., Cassivi, S. D., Schild, S. E., & Adjei, A. A. (2008). Non‐small cell lung cancer: Epidemiology, risk factors, treatment, and survivorship. Mayo Clinic Proceedings, 83, 584–594. 18452692 10.4065/83.5.584 PMC2718421

[jev212143-bib-0035] Nakanishi, M., & Rosenberg, D. W. (2013). Multifaceted roles of PGE2 in inflammation and cancer. Seminars in Immunopathology, 35, 123–137. 22996682 10.1007/s00281-012-0342-8 PMC3568185

[jev212143-bib-0036] Penfornis, P., Vallabhaneni, K. C., Whitt, J., & Pochampally, R. (2016). Extracellular vesicles as carriers of microRNA, proteins and lipids in tumor microenvironment. International Journal of Cancer, 138, 14–21. 25559768 10.1002/ijc.29417 PMC4924539

[jev212143-bib-0037] Peng, H., Wang, J., Li, J., Zhao, M., Huang, S. K., Gu, Y. Y., Li, Y., Sun, X. J., Yang, L., Luo, Q., & Huang, C. Z. (2016). A circulating non‐coding RNA panel as an early detection predictor of non‐small cell lung cancer. Life Sciences, 151, 235–242. 26946307 10.1016/j.lfs.2016.03.002

[jev212143-bib-0038] Pfaffl, M. W. (2001). A new mathematical model for relative quantification in real‐time RT‐PCR. Nucleic Acids Res., 29, e45. 11328886 10.1093/nar/29.9.e45 PMC55695

[jev212143-bib-0039] Prima, V., Kaliberova, L. N., Kaliberov, S., Curiel, D. T., & Kusmartsev, S. (2017). COX2/mPGES1/PGE2 pathway regulates PD‐L1 expression in tumor‐associated macrophages and myeloid‐derived suppressor cells. PNAS, 114, 1117–1122. 28096371 10.1073/pnas.1612920114 PMC5293015

[jev212143-bib-0040] Rana, S., Yue, S., Stadel, D., & Zöller, M. (2012). Toward tailored exosomes: The exosomal tetraspanin web contributes to target cell selection. International Journal of Biochemistry & Cell Biology, 44, 1574–1584. 22728313 10.1016/j.biocel.2012.06.018

[jev212143-bib-0041] Raposo, G., & Stoorvogel, W. (2013). Extracellular vesicles: exosomes, microvesicles, and friends. Journal of Cell Biology, 200, 373–383. 10.1083/jcb.201211138 PMC3575529 23420871

[jev212143-bib-0042] Reis, P. P., Drigo, S. A., Carvalho, R. F., Lopez Lapa, R. M., Felix, T. F., Patel, D., Cheng, D., Pintilie, M., Liu, G., & Tsao, M. S. (2020). Circulating miR‐16‐5p, miR‐92a‐3p, and miR‐451a in plasma from lung cancer patients: potential application in early detection and a regulatory role in tumorigenesis pathways. Cancers (Basel), 12. 10.3390/cancers12082071 PMC7465670 32726984

[jev212143-bib-0043] Salvi, V., Vaira, X., Gianello, V., Vermi, W., Bugatti, M., Sozzani, S., & Bosisio, D. (2016). TLR signalling pathways diverge in their ability to induce PGE2. Mediators Inflamm 2016, 5678046. 10.1155/2016/5678046 PMC5007370 27630451

[jev212143-bib-0044] Saul, M. J., Baumann, I., Bruno, A., Emmerich, A. C., Wellstein, J., Ottinger, S. M., Contursi, A., Dovizio, M., Donnini, S., Tacconelli, S., Raouf, J., Idborg, H., Stein, S., Korotkova, M., Savai, R., Terzuoli, E., Sala, G., Seeger, W., Jakobsson, P. J., … Steinhilber, D. (2019). miR‐574‐5p as RNA decoy for CUGBP1 stimulates human lung tumor growth by mPGES‐1 induction. Faseb Journal 33, 6933–6947. 30922080 10.1096/fj.201802547R

[jev212143-bib-0045] Schön, M. P., & Schön, M. (2008). TLR7 and TLR8 as targets in cancer therapy. Oncogene 27, 190–199. 18176600 10.1038/sj.onc.1210913

[jev212143-bib-0046] Shurtleff, M. J., Temoche‐Diaz, M. M., Karfilis, K. V., Ri, S., & Schekman, R. (2016). Y‐box protein 1 is required to sort microRNAs into exosomes in cells and in a cell‐free reaction. eLife, 5. 10.7554/eLife.19276 PMC5047747 27559612

[jev212143-bib-0047] Smith, W. L., Urade, Y., & Jakobsson, P. J. (2011). Enzymes of the cyclooxygenase pathways of prostanoid biosynthesis. Chem. Rev., 111, 5821–5865. 21942677 10.1021/cr2002992 PMC3285496

[jev212143-bib-0048] Sung, H., Ferlay, J., Siegel, R. L., Laversanne, M., Soerjomataram, I., Jemal, A., & Bray, F. (2021). Global cancer statistics 2020: GLOBOCAN estimates of incidence and mortality worldwide for 36 cancers in 185 countries. CA: A Cancer Journal for Clinicians, 71, 209–249. 33538338 10.3322/caac.21660

[jev212143-bib-0049] Temoche‐Diaz, M. M., Shurtleff, M. J., Nottingham, R. M., Yao, J., Fadadu, R. P., Lambowitz, A. M., & Schekman, R. (2019). Distinct mechanisms of microRNA sorting into cancer cell‐derived extracellular vesicle subtypes. eLife, 8, e47544. 10.7554/eLife.47544 31436530 PMC6728143

[jev212143-bib-0050] Thery, C., Witwer, K. W., Aikawa, E., Alcaraz, M. J., Anderson, J. D., Andriantsitohaina, R., Antoniou, A., Arab, T., Archer, F., Atkin‐Smith, G. K., Ayre, D. C., Bach, J. M., Bachurski, D., Baharvand, H., Balaj, L., Baldacchino, S., Bauer, N. N., Baxter, A. A., Bebawy, M., … Zuba‐Surma E.K. (2018). Minimal information for studies of extracellular vesicles 2018 (MISEV2018): a position statement of the International Society for Extracellular Vesicles and update of the MISEV2014 guidelines. Journal of Extracellular Vesicles, 7, 1535750. 30637094 10.1080/20013078.2018.1535750 PMC6322352

[jev212143-bib-0051] Thery, C., Zitvogel, L., & Amigorena, S. (2002). Exosomes: composition, biogenesis and function. Nature Reviews Immunology, 2, 569–579. 10.1038/nri855 12154376

[jev212143-bib-0052] Tian, F., Wang, J., Ouyang, T., Lu, N., Lu, J., Shen, Y., Bai, Y., Xie, X., & Ge, Q. (2019). MiR‐486‐5p Serves as a Good Biomarker in Nonsmall Cell Lung Cancer and Suppresses Cell Growth With the Involvement of a Target PIK3R1. Frontiers in Genetics, 10, 688. 31402930 10.3389/fgene.2019.00688 PMC6675869

[jev212143-bib-0053] Villarroya‐Beltri, C., Gutierrez‐Vazquez, C., Sanchez‐Cabo, F., Perez‐Hernandez, D., Vazquez, J., Martin‐Cofreces, N., Martinez‐Herrera, D. J., Pascual‐Montano, A., Mittelbrunn, M., & Sanchez‐Madrid, F. (2013). Sumoylated hnRNPA2B1 controls the sorting of miRNAs into exosomes through binding to specific motifs. Nature Communications, 4, 2980. 10.1038/ncomms3980 PMC3905700 24356509

[jev212143-bib-0054] Wang, D., & Dubois, R. N. (2010). Eicosanoids and cancer. Nature Reviews Cancer, 10, 181–193. 20168319 10.1038/nrc2809 PMC2898136

[jev212143-bib-0055] Yoshimatsu, K., Altorki, N. K., Golijanin, D., Zhang, F., Jakobsson, P. J., Dannenberg, A. J., & Subbaramaiah, K. (2001). Inducible prostaglandin E synthase is overexpressed in non‐small cell lung cancer. Clinical Cancer Research, 7, 2669–2674. 11555578

[jev212143-bib-0056] Zambrano, S., Bianchi, M. E., & Agresti, A. (2014). High‐throughput analysis of NF‐κB dynamics in single cells reveals basal nuclear localization of NF‐κB and spontaneous activation of oscillations. Plos One, 9, e90104. 24595030 10.1371/journal.pone.0090104 PMC3942427

[jev212143-bib-0057] Zhang, W., Thevapriya, S., Kim, P. J., Yu, W. P., Je, H. S., Tan, E. K., & Zeng, L. (2014). Amyloid precursor protein regulates neurogenesis by antagonizing miR‐574‐5p in the developing cerebral cortex. Nature Communications, 5, 3330. 10.1038/ncomms4330 24584353

[jev212143-bib-0058] Zheng, H., Zhan, Y., Liu, S., Lu, J., Luo, J., Feng, J., & Fan, S. (2018). The roles of tumor‐derived exosomes in non‐small cell lung cancer and their clinical implications. Journal of Experimental & Clinical Cancer Research, 37, 226. 30217217 10.1186/s13046-018-0901-5 PMC6137883

