# Supplementary material for: Small extracellular vesicle‐derived miR‐574‐5p regulates PGE2‐biosynthesis via TLR7/8 in lung cancer
Source: J Extracell Vesicles. 2021 Oct 1;10(12):12143. doi: 10.1002/jev2.12143 (PMC8485338; doi:10.1002/jev2.12143)
Supplement: Supplementary file 1 — Supporting Information [file JEV2-10-12143-s001.docx]

**Small extracellular vesicle-derived miR-574-5p regulates PGE_2_-biosynthesis via TLR7/8 in lung cancer**

Julia Donzelli ^1†^, Eva Proestler^1†^, Anna Riedel ^1^, Sheila Nevermann ^1^, Brigitte Hertel ^1^, Andreas Guenther ^2^, Stefan Gattenlöhner ^3^, Rajkumar Savai ^2,4,5^, Karin Larsson ^6^, Meike J. Saul ^1*^

^1^ Department of Biology, Technische Universität Darmstadt, Darmstadt, Germany

^2^ Department of Internal Medicine, Member of the German Center for Lung Research (DZL), Member of Cardio-Pulmonary Institute (CPI), Justus Liebig University, Giessen, Germany

^3^ Department of Pathology, Justus Liebig University, Giessen, Germany

^4^ Department of Lung Development and Remodeling, Member of the DZL, Member of CPI, Max Planck Institute for Heart and Lung Research, Bad Nauheim, Germany

^5^ Lung Microenvironmental Niche in Cancerogenesis, Institute for Lung Health (ILH), Justus Liebig University, Giessen, Germany

^6^ Rheumatology Unit, Department of Medicine, Karolinska University Hospital, Stockholm, Sweden

^†^ The authors contributed equally to this work.

* Correspondence author: Meike J. Saul ([saul@bio.tu-darmstadt.de](mailto:saul@bio.tu-darmstadt.de)),

Schnittspahnstraße 10, 64287 Darmstadt

**Figure S1**

**
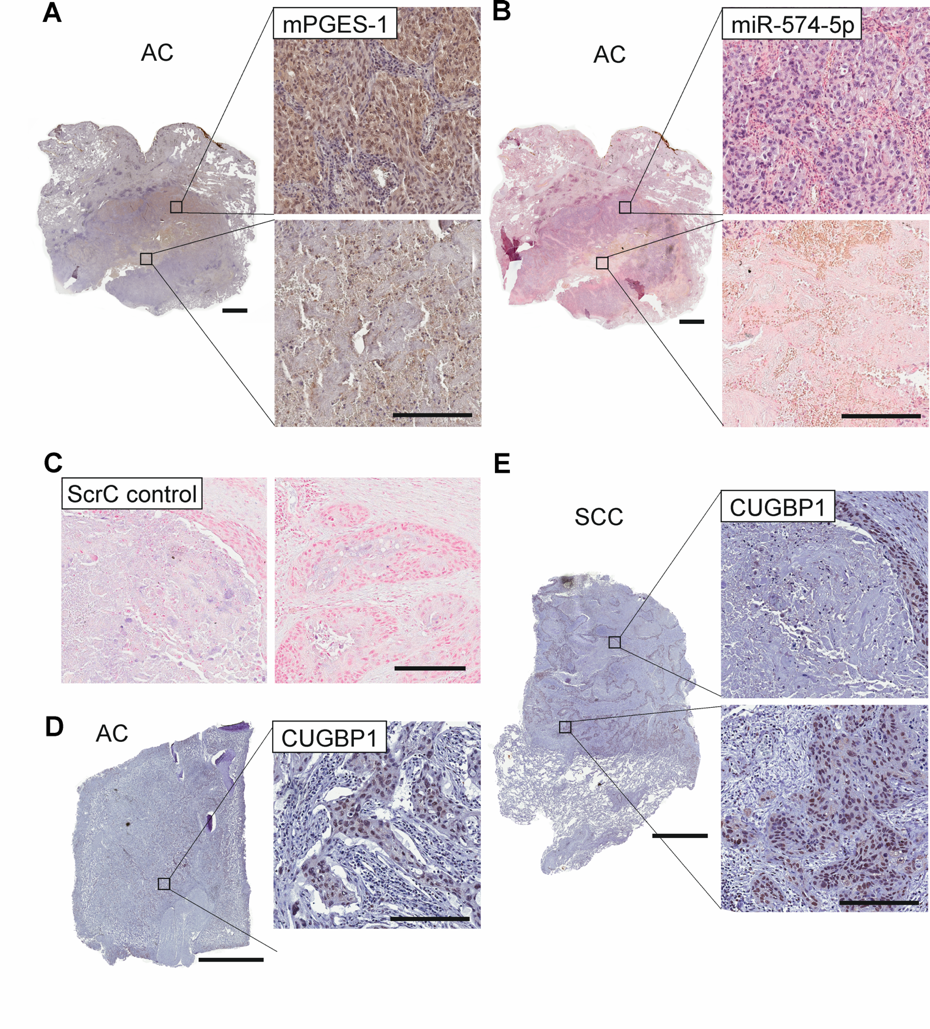
**

**Figure S1:** (A, B) MPGES-1 immunohistochemistry (IHC) staining and *in situ* hybridization (ISH) of AC lung tumor sections showing necrotic areas. MPGES-1 staining (brown) was counterstained with hematoxylin (blue). ISH was performed using a miR-574-5p-probe (blue) and sections were then counterstained with nuclear fast red (red). Scale bars: 2 mm, magnified images: 200 µm. (C) Negative control ISH of human SCC tissue using a ScrC probe (blue). The tissue was counterstained with nuclear fast red (red). Scale bar: 200 µm. (D, E) CUGBP1 staining in AC and SCC lung tumor sections. CUGBP1 (brown) was stained using IHC. Tissue sections were counterstained with hematoxylin (blue). Scale bars in AC tissue sections: 3 mm, SCC tissue sections: 2 mm, magnified images: 200 µm.

**Figure S2**


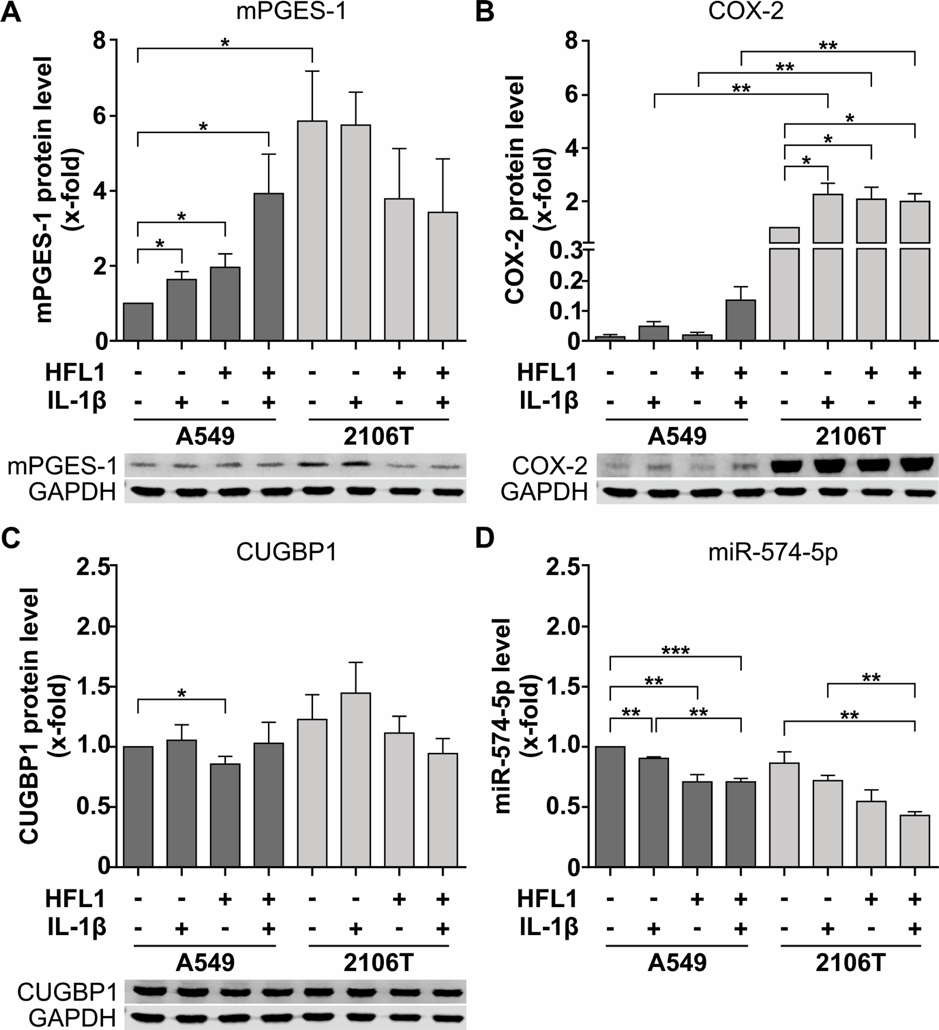


**Figure S2:** (A-C) Western blot analysis of mPGES-1, COX-2, and CUGBP1 in A549 and 2106T spheroid cultures. A549 and 2106T monoculture or co-culture spheroids with lung fibroblasts (HFL1) were generated with the hanging drop method. Spheroids were stimulated with 5 ng/ml IL-1β for 24 h. MPGES-1 and CUGBP1 levels were normalized to GAPDH and untreated A549 monoculture spheroids (AC: N=7, SCC: N=7). COX-2 levels were normalized to GAPDH and untreated 2106T monoculture spheroids (AC: N=3, SCC: N=5). (D) RT-qPCR analysis of intracellular miR-574-5p in A549 and 2106T spheroid cultures. MiR-574-5p levels were normalized to spike-in control ath-miR-159a and untreated A549 monoculture spheroids (A549: N=3, 2106T N=4). Results are shown as mean +SEM. Statistical differences between samples of the same cell type were determined by unpaired t-test, *p≤0.05; **p≤0.01; ***p≤0.001. Statistical differences between A549 and 2106T spheroids were determined by one-way ANOVA, *p≤0.05; **p≤0.01.

**Figure S3**

**
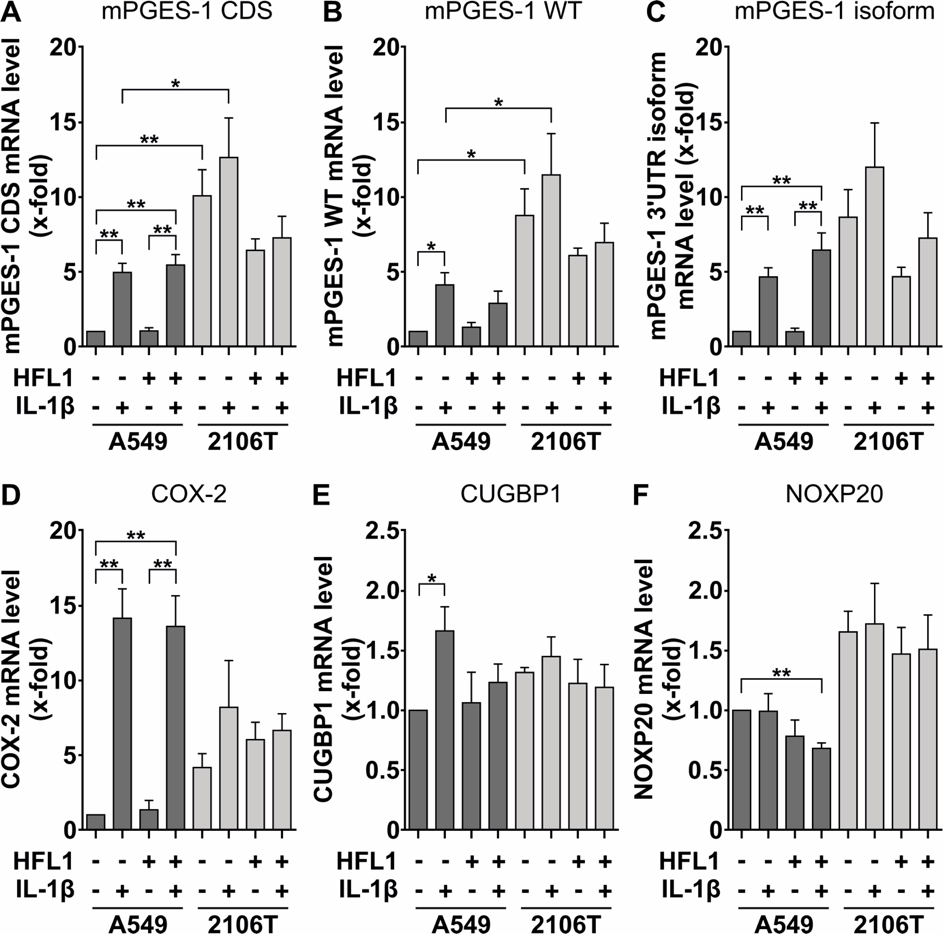
**

**Figure S3:** (A-F) RT-qPCR analysis of mPGES-1 CDS, mPGES-1 WT, mPGES-1 isoform, COX-2, CUGBP1, and NOXP20 mRNA. A549 and 2106T cells were cultivated in monoculture or co-culture spheroids with lung fibroblasts (HFL1), which were generated with the hanging drop method. Spheroids were stimulated with 5 ng/ml IL-1β for 24 h. MRNA levels were normalized to GAPDH and untreated A549 monoculture spheroids (A549: N=3, 2106T: N=4). Results are shown as mean +SEM. Statistical differences between samples of the same cell-type were determined by unpaired t-test, *p≤0.05; **p≤0.01; ***p≤0.001; ****p≤0.0001. Statistical differences between A549 and 2106T spheroids were determined by one-way ANOVA, *p≤0.05; **p≤0.01.

**Figure S4**

**
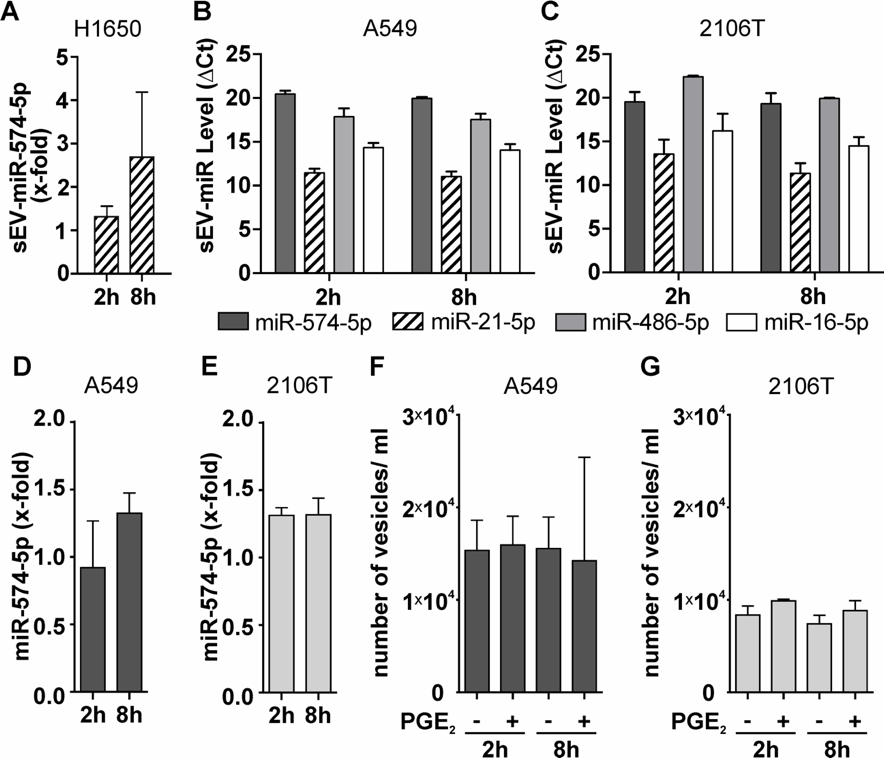
**

**Figure S4:** (A) SEV level of miR-574-5p isolated from H1650 cell culture supernatants. H1650 cells were stimulated with 5 nM PGE_2_ for 2 h and 8 h prior to supernatant harvesting. MiR levels were analyzed by RT-qPCR, normalized to the spike-in control ath-miR-159a and folded to their corresponding control (N=4). Data are shown as mean +SEM. (B, C) RT-qPCR analysis of sEV-derived miR-574-5p, miR-21-5p, miR-486-5p, and miR-16-5p from A549 and 2106T cell culture supernatant after 2 h and 8 h. The relative expression of each miR is shown as the ∆Ct value, normalized to the spike-in control ath-miR-159a. In 2106T cells, miR-486-5p was not detectable (N=3). (D, E) RT-qPCR analysis of intracellular miR-574-5p in A549 or 2106T cells after PGE_2_ stimulation. For A549 samples, miR-574-5p levels were normalized to miRTC and untreated samples (N=3). For 2106T samples, miR-574-5p levels were normalized to spike-in control ath-miR-159a and untreated samples (N=3). (F, G) Number of A549- or 2106T -derived sEV per ml after PGE_2_ stimulation. SEV numbers were determined using a Zetasizer Nano S (Malvern Panalytical) in unpurified cell culture supernatants (N=4).

**Figure S5**


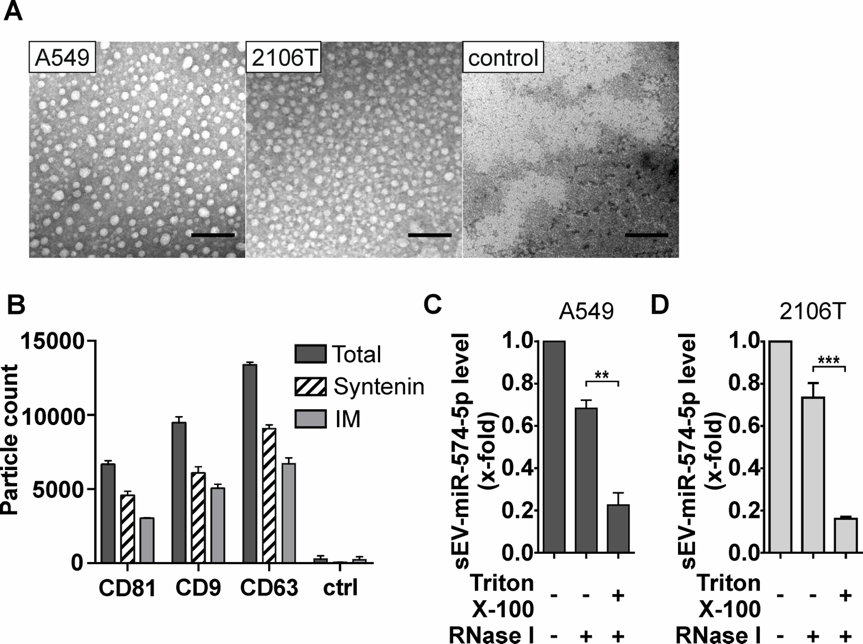


**Figure S5:** (A) A549- and 2106T-derived miR-574-5p oe sEV were purified using differential ultracentrifugation. The purification of sEV was proven via transmission electron microscopy (TEM). Scale bars: 100 nm. (B) Particle count after ultracentrifugation. MiR-574-5p oe sEV from 1 ml 2106T cell supernatant were isolated and analyzed with the ExoView R100^TM^ platform by interferometry (IM) vesicle sizing and single sEV cargo staining of Syntenin. IM measurement detected vesicles within a range of 50 – 200 nm. A total particle count of 29,796 particles per ml was determined. (C, D) MiR-574-5p oe sEV from A549 and 2106T cells were treated with RNase with and without Triton X-100. MiR-574-5p levels were determined via RT-qPCR and normalized to the spike-in control ath-miR-159a. Relative changes to untreated sEV are shown as mean +SEM (N=3), t-test **p≤0.01; ***p≤0.001.

**Figure S6**


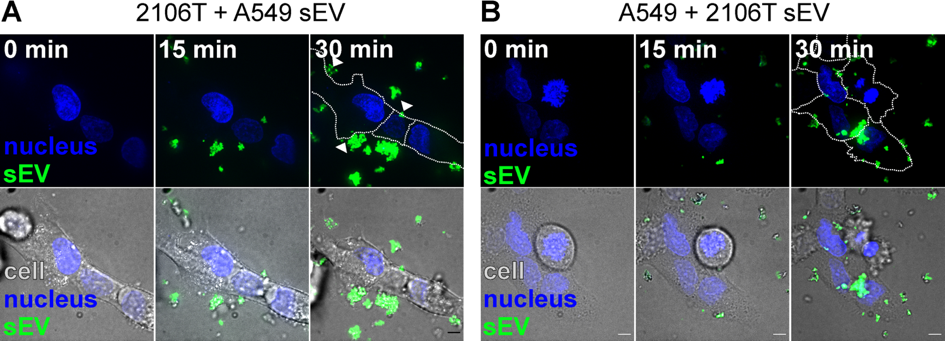


**Figure S6:** (A, B) Live-cell imaging of sEV uptake in A549 and 2106T cells. Cells were treated with sEV isolated from the other cell line. Cells were visualized using differential interference contrast (DIC, gray) and nuclear staining with 5 µg/ml Hoechst 33258 (blue). SEV were stained with the lipophilic tracer 3,3′-dioctadecyloxacarbocy-anine perchlorate DIO (green). SEV uptake was imaged over 30 min. 2106T cells take up A549-derived sEV at a slower rate and smaller number than A549 cells take up 2106T-derived sEV. SEV accumulate at the cell membranes (white arrows). Representative images of three independent biologicals replicates with at least three technical replicates are shown. Scale bars: 10 µm.

**Figure S7**

**
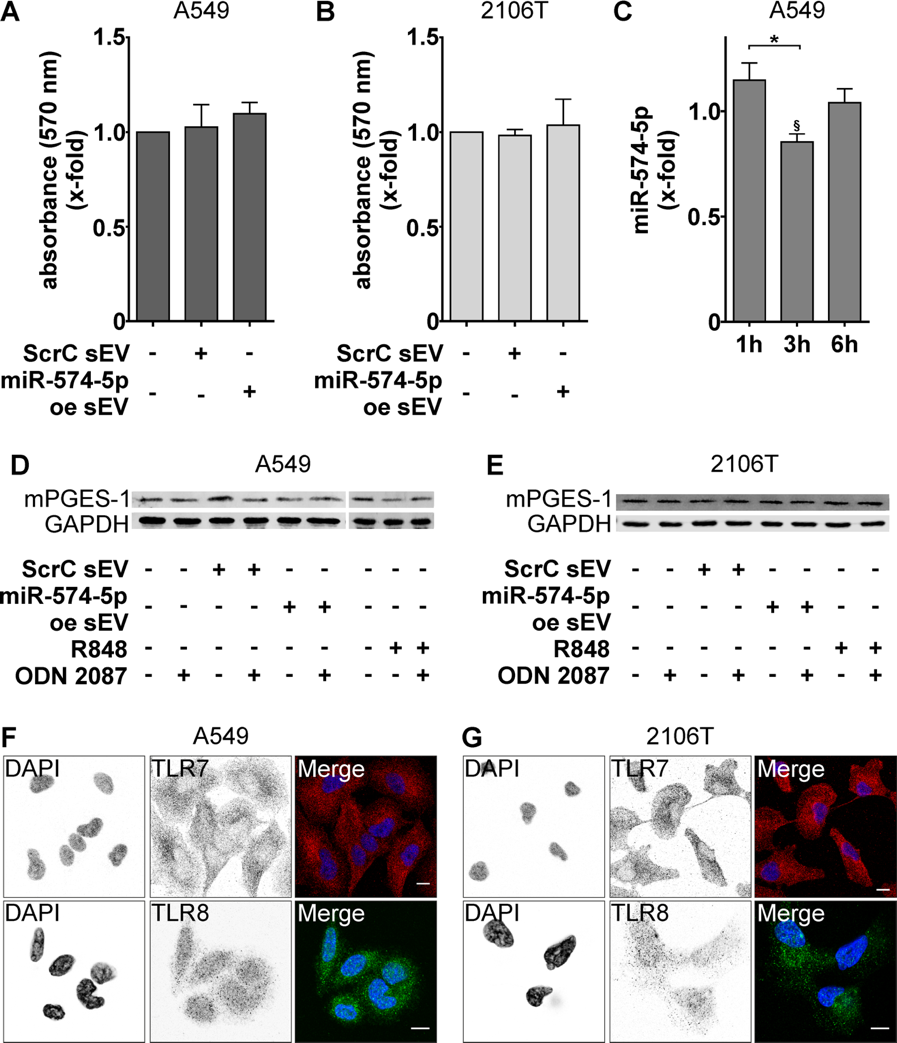
**

**Figure S7:** (A, B) Tetrazolium reduction assay of A549 or 2106T cells after treatment with 2 µg/ml miR-574-5p oe or ScrC sEV. Effects on cell proliferation or metabolism were assessed with 3-(4,5-dimethylthiazol-2-yl)-2,5-diphenyltetrazoliumbromide (MTT, N=3). Results are shown as mean +SEM. (C) RT-qPCR analysis of intracellular miR-574-5p in A549 cells after stimulation with miR-574-5p oe sEV. MiR-574-5p levels were normalized to spike-in control ath-miR-159a and untreated samples (N=3). Results are shown as mean +SEM, unpaired t-test to other samples, *p≤0.05. Unpaired t-test to untreated control § p≤0.05. (D, E) MPGES-1 Western blot analysis of A549 or 2106T cells treated with 2 µg/ml sEV, 100 ng/ml R848 (TLR7/8 ligand) or 200 mM ODN 2088 Control (ODN 2087) (TLR7/8 antagonist) for 24 h. (F, G) TLR7 (red) and TLR8 (green) staining in A549 and 2106T cells. Nuclei were visualized with DAPI (blue). Shown are representative images of three independent experiments. Scale bars: 10 µm.

**Table S1:** Overview of the experimental setup of this study to ensure reproducibility according to the MISEV2018 guidelines.

| Major recommendations of MISEV2018 guidelines | Experimental protocols in this study |
| --- | --- |
| Nomenclature | Extracellular vesicles are named small extracellular vesicles “sEV” according to the physical characteristic for extracellular vesicles < 100 nm |
| Cell culture conditions | All information on cell culture conditions, medium composition and preparation methods are provided and are in line with the MISEV2018 guidelines |
| Method of enrichment | sEV isolation via differential ultracentrifugation is described in detail in the material and method section |
| Quantification | sEV source and sEV concentrations for each experiment are described, total particle number is provided |
| General characterization | Surface proteins and size distribution were characterized with the ExoView R100^TM^ platform |
| Characterization of single vesicles | Single vesicles were characterized with TEM, the ExoView R100^TM^ platform and live-cell imaging |
| Topology of functional molecules | Topology of miR-574-5p was confirmed with RNase digestion and detergent permeabilization |
